# Development of a multi-antigenic SARS-CoV-2 vaccine candidate using a synthetic poxvirus platform

**DOI:** 10.1038/s41467-020-19819-1

**Published:** 2020-11-30

**Authors:** Flavia Chiuppesi, Marcela d’Alincourt Salazar, Heidi Contreras, Vu H. Nguyen, Joy Martinez, Yoonsuh Park, Jenny Nguyen, Mindy Kha, Angelina Iniguez, Qiao Zhou, Teodora Kaltcheva, Roman Levytskyy, Nancy D. Ebelt, Tae Hyuk Kang, Xiwei Wu, Thomas F. Rogers, Edwin R. Manuel, Yuriy Shostak, Don J. Diamond, Felix Wussow

**Affiliations:** 1 Department of Hematology and Transplant Center, City of Hope National Medical Center, Duarte, CA 91010 USA; 2 Department of Immuno-Oncology, Beckman Research Institute of the City of Hope, Duarte, CA 91010 USA; 3 Integrative Genomics Core, Beckman Research Institute of the City of Hope, Duarte, CA 91010 USA; 4 Division of Infectious Diseases and Global Public Health, University of California San Diego, School of Medicine, 9500 Gilman Dr, La Jolla, CA 92093 USA; 5 Scripps Research, Department of Immunology and Microbiology, 10550N Torrey Pines Rd, La Jolla, CA 92037 USA; 6 Research Business Development, City of Hope, Duarte, CA 91010 USA

**Keywords:** Live attenuated vaccines, SARS-CoV-2, Viral infection

## Abstract

Modified Vaccinia Ankara (MVA) is a highly attenuated poxvirus vector that is widely used to develop vaccines for infectious diseases and cancer. We demonstrate the construction of a vaccine platform based on a unique three-plasmid system to efficiently generate recombinant MVA vectors from chemically synthesized DNA. In response to the ongoing global pandemic caused by SARS coronavirus-2 (SARS-CoV-2), we use this vaccine platform to rapidly produce fully synthetic MVA (sMVA) vectors co-expressing SARS-CoV-2 spike and nucleocapsid antigens, two immunodominant antigens implicated in protective immunity. We show that mice immunized with these sMVA vectors develop robust SARS-CoV-2 antigen-specific humoral and cellular immune responses, including potent neutralizing antibodies. These results demonstrate the potential of a vaccine platform based on synthetic DNA to efficiently generate recombinant MVA vectors and to rapidly develop a multi-antigenic poxvirus-based SARS-CoV-2 vaccine candidate.

## Abstract (web summary)

Chiuppesi *et al*. demonstrate the use of a synthetic poxvirus-based platform to rapidly generate multi-antigenic vaccine candidates expressing spike and nucleocapsid antigens of SARS-CoV-2. Immunization of mice stimulates potent antigen-specific humoral and cellular immune responses, including neutralizing antibodies.

## Introduction

Modified Vaccinia Ankara (MVA) is a highly attenuated poxvirus vector that is widely used to develop vaccine approaches for infectious diseases and cancer^1–3^. As a result of the attenuation process through 570 virus passages on chicken embryo fibroblast (CEF), MVA has acquired multiple major and minor genome alterations^4,5^, leading to severely restricted host cell tropism^6^. MVA can efficiently propagate in CEF and a baby hamster kidney (BHK) cell line, while in most mammalian cells, including human cells, MVA replication is limited due to a late block in virus assembly^3,6^. Its excellent safety and immunogenicity profile in addition to its versatile expression system and large capacity to incorporate heterologous DNA make MVA an ideal vector for recombinant vaccine development^1,7^. We developed various MVA vaccine candidates for animal models of cytomegalovirus-associated disease in pregnant women while demonstrating vaccine efficacy in several clinical trials in solid tumor and stem cell transplant patients^8–13^.

Since the outbreak of the novel severe acute respiratory syndrome coronavirus-2 (SARS-CoV-2) in December 2019^14,15^, the virus has spread to more than 200 countries worldwide, causing a pandemic of global magnitude with over a million deaths. Many vaccine candidates are currently under rapid development to control this global pandemic^16–18^, some of which have entered into clinical trials with unprecedented pace^17,19^. Most of these approaches employ antigenic forms of the Spike (S) protein as it is considered the primary target of protective immunity^16,20–22^. The S protein mediates SARS-CoV-2 entry into a host cell through binding to angiotensin-converting enzyme 2 (ACE) and is the major target of neutralizing antibodies (NAb)^23–25^. Studies in rhesus macaques show that vaccine strategies based on the S antigen can prevent SARS-CoV-2 infection in this relevant animal model^18^, indicating that the S antigen may be sufficient as a vaccine immunogen to elicit SARS-CoV-2 protective immunity. However, a recent study showed that even patients without measurable NAb can recover from SARS-CoV-2 infection, suggesting that protection against SARS-CoV-2 infection is mediated by both humoral and cellular immunity to multiple immunodominant antigens, including S and nucleocapsid (N) antigens^20,26,27^.

We developed a vaccine platform based on a uniquely designed three-plasmid system to efficiently generate recombinant MVA vectors from chemically synthesized DNA. In response to the ongoing global pandemic caused by SARS-CoV-2, we used this vaccine platform to rapidly produce synthetic MVA (sMVA) vectors co-expressing full-length S and N antigens. We demonstrate that these sMVA vectors stimulate robust SARS-CoV-2 antigen-specific humoral and cellular immunity in mice, including potent NAb. These results emphasize the value of a vaccine platform based on synthetic DNA to efficiently produce recombinant poxvirus vectors and warrant further pre-clinical and clinical testing of a multi-antigenic sMVA vaccine candidate to control the ongoing SARS-CoV-2 pandemic and its devastating consequences.

## Results

### Construction of sMVA

**Fig. 1:**
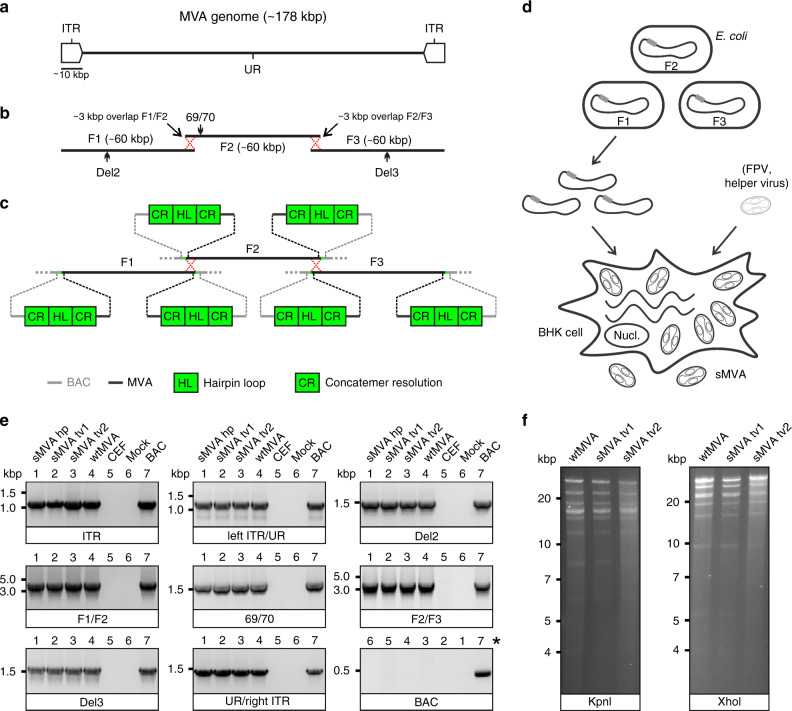
sMVA construction and characterization. **a** Schematic of MVA genome. The MVA genome is ~178 kbp in length and contains an internal unique region (UR) flanked by ~9.6 kbp inverted terminal repeat (ITR) sequences. **b** sMVA fragments. The three sub-genomic sMVA fragments (F1–F3) comprise ~60 kbp of the left, central, and right part of the MVA genome as indicated. sMVA F1/F2 and F2/F3 share ~3 kbp overlapping homologous sequences for recombination (red dotted crossed lines). Approximate genome positions of commonly used MVA insertion sites (Del2, IGR69/70, Del3) are indicated. **c** Terminal CR/HL/CR sequences. Each of the sMVA fragments contains at both ends a sequence composition comprising a duplex copy of the MVA terminal hairpin loop (HL) flanked by concatemeric resolution (CR) sequences. BAC bacterial artificial chromosome vector. **d** sMVA reconstitution. The sMVA fragments are maintained in *E. coli*, isolated from the bacteria, and co-transfected into BHK cells, which are subsequently infected with FPV as a helper virus to initiate sMVA virus reconstitution. **e** PCR analysis. CEFs infected with sMVA, derived with either FPV HP1.441 (sMVA hp) or with FPV TROVAC from two independent virus reconstitutions (sMVA tv1 and sMVA tv2), were investigated by PCR for several genome positions, including the ITR sequences, the transition from the left or right ITR into the internal UR (left ITR/UR; UR/right ITR), the Del2, IGR69/70 and Del3 insertion sites, and the F1/F2 and F2/F3 recombination sites. The removal of the BAC vector sequences was also investigated. PCR with wtMVA-infected and uninfected cells, without sample (mock), or with MVA BAC was performed as controls. *Note the different sample order for the BAC-specific PCR as indicated by numbers. **f** Restriction pattern analysis. Viral DNA isolated from ultra-purified sMVA (sMVA tv1 and sMVA tv2) or wtMVA virus was compared by KpnI and XhoI restriction enzyme digestion. Experiments in **e** and **f** were performed twice with similar results.

To develop the three-plasmid system of the sMVA vaccine platform, we designed three unique synthetic sub-genomic MVA fragments (sMVA F1–F3) based on the MVA genome sequence published by Antoine et al.^4^, which is ~178 kbp in length and contains ~9.6 kbp inverted terminal repeats (ITRs) (Fig. 1a). The three fragments were designed as follows: sMVA F1 comprises ~60 kbp of the left part of the MVA genome, including the left ITR sequences; sMVA F2 contains ~60 kbp of the central part of the MVA genome; and sMVA F3 contains ~60 kbp of the right part of the MVA genome, including the right ITR sequences (Fig. 1b). sMVA F1 and F2 as well as sMVA F2 and F3 were designed to share ~3 kb overlapping homologous sequences to promote recombination of the three sMVA fragments (Fig. 1b). In addition, a duplex copy of the 165-nucleotide-long MVA terminal hairpin loop (HL) flanked by concatemeric resolution (CR) sequences was added to both ends of each of the three sMVA fragments (Fig. 1c). Such CR/HL/CR sequence arrangements are formed at the genomic junctions in poxvirus DNA replication intermediates and are essential for genome resolution and packaging^28–32^. When circular DNA plasmids containing these CR/HL/CR sequences are transfected into helper virus-infected cells, they spontaneously resolve into linear minichromosomes with intact terminal HL sequences^29,30,33^. Based on these findings, we hypothesized that the three sMVA fragments as shown in Fig. 1b, c, when co-transfected as circular DNA plasmids into helper virus-infected cells, resolve into linear minichromosomes, recombine with each other via the homologous sequences, and are ultimately packaged as full-length genomes into sMVA virus particles. The three sMVA fragments were cloned in *Escherichia coli* as bacterial artificial chromosome (BAC) clones. Sequence analysis confirmed the identity of the reference sequences of all three BAC-cloned sMVA fragments deposited in NCBI.

Using a previously employed procedure to rescue MVA from a BAC^8,9,34^, sMVA virus was reconstituted with Fowl pox (FPV) as a helper virus upon co-transfection of the three DNA plasmids into BHK cells (Fig. 1d), which are non-permissive for FPV^35^. Two different FPV strains (HP1.441 and TROVAC)^36,37^ were used to promote sMVA virus reconstitution. Purified sMVA virus stocks were produced following virus propagation in CEF, which are commonly used for MVA vaccine production. The virus titers achieved with reconstituted sMVA virus were similar to virus titers achieved with wild-type MVA (wtMVA) (Table S1).

### In vitro characterization of sMVA

To characterize the viral DNA of sMVA, DNA extracts from sMVA and wtMVA-infected CEF were compared for several MVA genome positions by PCR. Similar PCR results were obtained with sMVA and wtMVA for all evaluated genome positions (Fig. 1e), including the F1/F2 and F2/F3 recombination sites, indicating efficient recombination of the three sMVA fragments. Additional PCR analysis indicated the absence of BAC vector sequences in sMVA viral DNA (Fig. 1e), suggesting spontaneous and efficient removal of bacterial vector elements upon sMVA virus reconstitution. Comparison of viral DNA from purified sMVA and wtMVA virus by restriction enzyme digestion revealed a similar genome pattern between sMVA and wtMVA (Fig. 1f). Sequencing analysis of PCR products derived from sMVA viral DNA confirmed the MVA genome sequence at several positions, including the F1/F2 and F2/F3 recombination sites. Furthermore, whole-genome sequence analysis of one of the sMVA virus isolates reconstituted with FPV TROVAC confirmed the assembly of the reference MVA genome sequence and absence of BAC vector sequences in viral DNA originating from reconstituted sMVA virus.

**Fig. 2:**
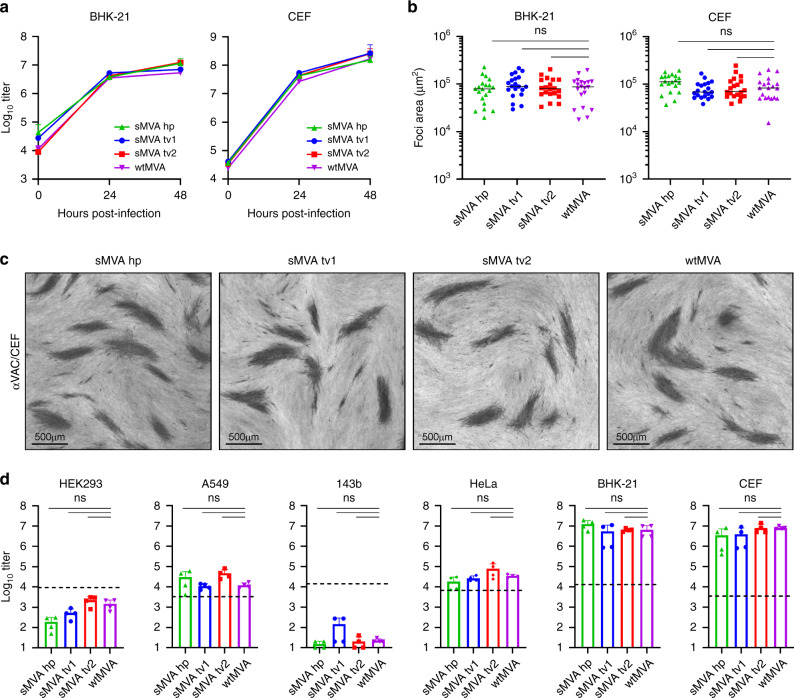
sMVA replication properties. The replication properties of sMVA, derived either with FPV HP1.441 (sMVA hp) or with FPV TROVAC from two independent sMVA virus reconstitutions (sMVA tv1 and sMVA tv2), were compared with that of wtMVA. **a** Replication kinetics. BHK or CEF cells were infected in triplicates (*n* = 3) for each time point at 0.02 multiplicity of infection (MOI) with sMVA or wtMVA and viral titers of the inoculum and each triplicate infection were determined at 24 and 48 h post infection on CEF. Mixed-effects model with the Geisser-Greenhouse correction followed by Tukey’s multiple comparison test were applied; at 24 and 48 h post-infection differences between groups were not significant (*p* > 0.05). **b**, **c** Viral foci size analysis. BHK or CEF cell monolayers were infected at 0.002 MOI with sMVA or wtMVA and areas of viral foci (*n* = 20) were determined at 24 h post infection following immunostaining with anti-Vaccinia polyclonal antibody (αVAC). Panel **c** provides examples of sMVA and wtMVA viral foci following immunostaining of infected CEF. **d** Host cell range analysis. Various human cell lines (HEK293, A549, 143b, and HeLa), CEF or BHK cells were infected in duplicates (*n* = 2) at 0.01 MOI with sMVA or wtMVA and virus titers of each duplicate infection were determined in duplicates (*n* = 4 in total) at 48 h post infection on CEF. Dotted lines indicate the calculated virus titer of the inoculum based on 0.01 MOI. Differences between groups in **b** and **d** were calculated using one-way ANOVA followed by Tukey’s (**b**) or Dunnett’s (**d**) multiple comparison tests; ns = not significant (*p* > 0.05). Data in **a** and **d** are presented as mean values + SD. Lines in **b** represent median values. Source data are provided as a Source Data file.

To characterize the replication properties of sMVA, growth kinetics of sMVA and wtMVA were compared in BHK and CEF cells, two cell types known to support productive MVA replication^6^. This analysis revealed similar growth kinetics of sMVA and wtMVA in both BHK and CEF cells (Fig. 2a). In addition, similar areas of viral foci were determined in BHK and CEF cell monolayers infected with sMVA or wtMVA (Fig. 2b, c), suggesting similar capacity of sMVA and wtMVA to spread in MVA permissive cells. Compared to the productive replication of sMVA and wtMVA in BHK and CEF cells, only limited virus production was observed with sMVA or wtMVA following infection of various human cell lines (Fig. 2d). These results are consistent with the severely restricted replication properties of MVA and show that the sMVA virus can efficiently propagate in BHK and CEF cells, while it is unable to propagate in human cells.

### In vivo immunogenicity of sMVA

**Fig. 3:**
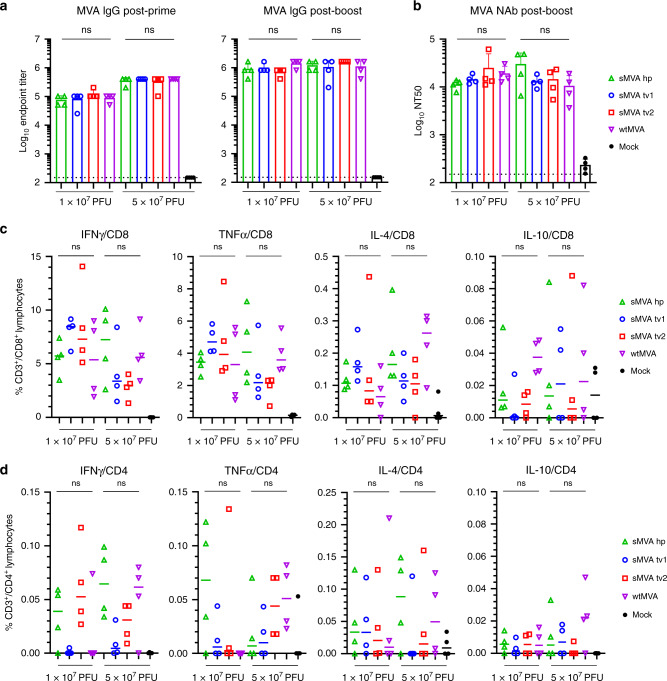
sMVA in vivo immunogenicity. sMVA derived either with FPV HP1.441 (sMVA hp) or with FPV TROVAC from two independent virus reconstitutions (sMVA tv1 and sMVA tv2) was compared by in vivo analysis with wtMVA. C57BL/6 mice (*n* = 4) were immunized twice at a 3-week interval with low (1 × 10^7^ PFU) or high (5 × 10^7^ PFU) dose of sMVA or wtMVA. Mock-immunized mice were used as controls. **a** Binding antibodies. MVA-specific binding antibodies (IgG titer) stimulated by sMVA or wtMVA were measured after the first and second immunization by ELISA. **b** NAb responses. MVA-specific NAb titers induced by sMVA or wtMVA were measured after the booster immunization against recombinant wtMVA expressing a GFP marker. **c**, **d** T-cell responses. MVA-specific IFNγ, TNFα, IL-4, and IL-10-secreting CD8+ (**c**) and CD4+ (**d**) T-cell responses induced by sMVA or wtMVA after two immunizations were measured by flow cytometry following ex vivo antigen stimulation using B8R immunodominant peptides. Differences between groups were evaluated using one-way ANOVA with Tukey’s multiple comparison test; ns = not significant (*p* > 0.05). Data in **a** and **b** are presented as mean values + SD. Lines in **c** and **d** represent median values. Source data are provided as a Source Data file.

To characterize sMVA in vivo, the immunogenicity of sMVA and wtMVA was compared in C57BL/6 mice following two immunizations at high or low dose. MVA-specific binding antibodies stimulated by sMVA and wtMVA after the first and second immunization were comparable (Figs. 3a and S1a). While the antibody levels in the high dose vaccine groups exceeded those of the low dose vaccine groups after the first immunization, similar antibody levels in the high and low dose vaccine groups were observed after the second immunization. In addition, no significant differences were detected in the levels of MVA-specific NAb responses induced by sMVA and wtMVA after the second immunization (Figs. 3b and S1b). MVA-specific T-cell responses determined after the booster immunization by ex vivo antigen stimulation using immunodominant peptides^38^ revealed similar MVA-specific T-cell levels in mice receiving sMVA or wtMVA (Figs. 3c, d and S1c, d). These results indicate that sMVA virus has a similar capacity to wtMVA in inducing MVA-specific humoral and cellular immunity in mice.

### Construction of sMVA SARS-CoV-2 vaccine vectors

**Fig. 4:**
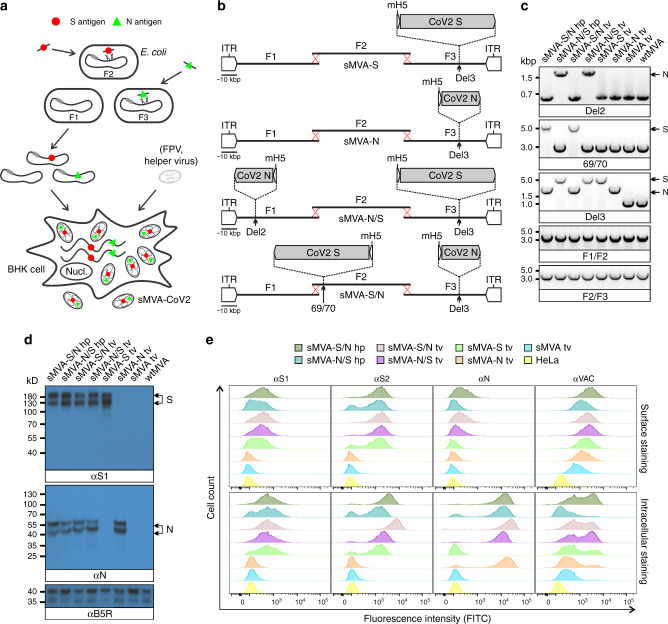
Construction and characterization of sMVA-CoV2 vectors. **a** Schematic representation of vector construction. S and N antigen sequences (red spheres and green triangles) were inserted into sMVA fragments F2 and F3 by bacterial recombination methods in *E. coli*. The modified sMVA fragments of F2 and F3 with inserted antigen sequences and the unmodified sMVA fragment F1 were isolated from *E. coli* and co-transfected into FPV-infected BHK cells to initiate virus reconstitution. **b** Schematics of single (sMVA-S, sMVA-N) and double (sMVA-N/S, sMVA-S/N) recombinant sMVA-CoV2 vectors with S and N antigen sequences inserted into commonly used MVA insertion sites (Del2, IGR69/70, Del3) as indicated. All antigens were expressed via the Vaccinia mH5 promoter. ITR inverted terminal repeat. **c** PCR analysis. CEFs infected with the single and double recombinant sMVA-CoV2 vectors derived with FPV HP1.441 (sMVA-S/N hp, sMVA-N/S hp) or TROVAC (sMVA-S/N tv, sMVA-N/S tv, sMVA-S tv, sMVA-N tv) were evaluated by PCR with primers specific for the Del2 and Del3 insertion sites harboring the N and S antigen sequences or primers specific for the F1/F2 and F2/F3 recombination sites. **d** Western Blot. BHK cells infected with the sMVA-CoV2 vectors were evaluated for antigen expression by Western Blot using anti-S1 and anti-N antibodies (αS1 and αN). Vaccinia B5R protein was verified as infection control. Higher and lower molecular weight bands may represent mature and immature protein species. **e** Flow cytometry staining. HeLa cells infected with the vaccine vectors were evaluated by cell surface and intracellular flow staining using anti-S1, S2, and N antibodies (αS1, αS2, and αN). Live cells were used to evaluate cell surface antigen expression. Fixed and permeabilized cells were used to evaluate intracellular antigen expression. Anti-Vaccinia virus antibody (αVAC) was used as staining control to verify MVA protein expression. Cells infected with sMVA or wtMVA or uninfected cells were used as controls for experiments in **c**, **d**, and **e** as indicated. The experiments in **c**, **d**, and **e** were performed twice with similar results.

Using highly efficient BAC recombination techniques in *E. coli*^39,40^, full-length SARS-CoV-2 S and N antigen sequences were inserted into commonly used MVA insertion sites located at different positions within the three sMVA fragments. Combinations of modified and unmodified sMVA fragments were subsequently co-transfected into FPV-infected BHK cells to reconstitute sMVA SARS-CoV-2 (sMVA-CoV2) vaccine vectors expressing the S and N antigen sequences alone or combined (Fig. 4a, b). In the single recombinant vectors encoding S or N alone, termed sMVA-S and sMVA-N, the antigen sequences were inserted into the Deletion 3 (Del3) site (Figs. 1b and 4b)^5^. In the double recombinant vectors encoding both S and N, termed sMVA-N/S and sMVA-S/N, the antigen sequences were inserted into Del3 and the Deletion 2 (Del2) site (sMVA-N/S), or they were inserted into Del3 and the intergenic region between MVA 069R and 070L (IGR69/70) (sMVA-S/N) (Figs. 1b and 4b)^5,41^. All antigen sequences were inserted into sMVA together with mH5 promoter to promote antigen expression during early and late phases of MVA replication^42,43^. sMVA-CoV2 vaccine vectors were reconstituted with FPV strains HP1.441 or TROVAC. Purified virus of the sMVA-CoV2 vectors produced using CEF reached titers that were comparable to those achieved with sMVA or wtMVA (Table S1). PCR and sequencing analysis of the Del2, Del3, and IGR69/70 MVA insertion sites confirmed the integrity and insertion of the SARS-CoV-2 antigen sequences in all sMVA-CoV2 vaccine vectors (Fig. 4c). Moreover, whole-genome sequencing of all double-recombinant sMVA-CoV2 vaccine vectors—reconstituted either with FPV strain TROVAC or HP1.441—verified the reference sequences of these vaccine constructs deposited in NCBI and confirmed the SARS-CoV-2 antigen sequences at the insertion sites, the identity of the MVA genome, and removal of the BAC vector sequences.

### In vitro characterization of sMVA-CoV2 vaccine vectors

To characterize S and N antigen expression by the sMVA-CoV2 vectors, BHK cells infected with sMVA-CoV2 vectors were evaluated by Immunoblot using S- and N-specific antibodies. This analysis confirmed the expression of the S or N antigen alone by the single recombinant vaccine vectors sMVA-S and sMVA-N, while the expression of both the S and N antigens was confirmed for the double recombinant vectors sMVA-N/S and sMVA-S/N (Fig. 4d).

Further characterization of the antigen expression by the sMVA-CoV2 vectors in HeLa cells using cell surface and intracellular flow cytometry staining confirmed single and dual S and N antigen expression by the single and double recombinant vaccine vectors. Staining with S-specific antibodies revealed abundant cell surface and intracellular antigen expression by all vectors encoding the S antigen (sMVA-S, sMVA-N/S, sMVA-S/N) (Fig. 4e). In contrast, staining with anti-N antibody revealed predominantly intracellular antigen expression by all vectors encoding the N antigen (sMVA-N, sMVA-N/S, sMVA-S/N) (Fig. 4e), although cell surface staining was also observed to a minor extent. S and N antigen expression by the sMVA-CoV2 vectors was also investigated by immunofluorescence imaging. This analysis confirmed co-expression of the S and N antigens by the double recombinant vaccine vectors and indicated efficient cell surface and intracellular expression of the S antigen, whereas the expression of the N antigen was predominantly observed intracellularly (Fig. S2a–c). Furthermore, immunofluorescence imaging in addition to intracellular flow cytometry by dual antibody staining demonstrated co-expression of the S and N antigens within the same cell by the double recombinant sMVA-CoV2 vectors (Fig. S2a–d). These results demonstrate efficient antigen expression by the single and double recombinant sMVA-CoV2 vectors.

### In vivo immunogenicity of sMVA-CoV2 vaccine vectors

**Fig. 5:**
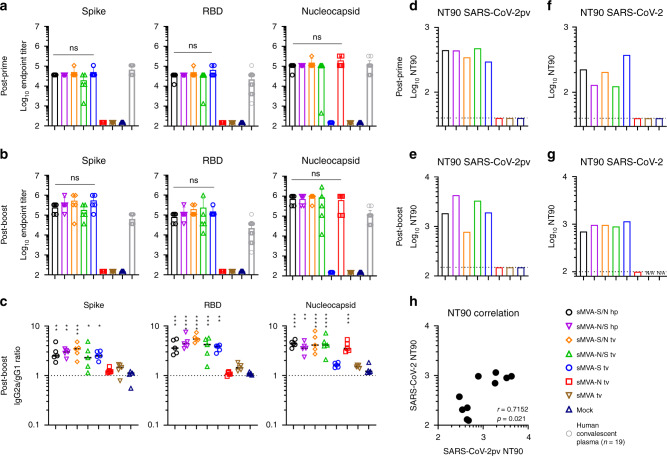
Humoral immune responses stimulated by sMVA-CoV2 vectors. Balb/c mice (*n* = 5) immunized twice in a 3-week interval with 5 × 10^7^ PFU of the single and double recombinant sMVA-CoV2 vectors derived with FPV HP1.441 (sMVA-S/N hp and sMVA-N/S hp) or TROVAC (sMVA-S/N tv, sMVA-N/S tv, sMVA-S tv, sMVA-N tv) were evaluated for SARS-CoV-2-specific humoral immune responses. **a**, **b** Binding antibodies. S-, RBD-, and N-specific binding antibodies induced by the vaccine vectors were evaluated after the first (**a**) and second (**b**) immunization by ELISA. As a comparison, antigen-specific endpoint titers measured in *n* = 19 plasma samples from SARS-CoV-2 convalescent individuals (Fig. S4) were added in **a** and **b**. Data are presented as mean values + SD. One-way ANOVA with Tukey’s multiple comparison test was used to evaluate the differences between binding antibody end-point titers. **c** IgG2a/IgG1 isotype ratio. S-, RBD-, and N-specific binding antibodies of the IgG2a and IgG1 isotype were measured after the second immunization using 1:10,000 serum dilution, and absorbance reading was used to calculate IgG2a/IgG1 antibody ratio. Lines represent median values. One-way ANOVA with Dunnett’s multiple comparison test was used to compare each group mean IgG2a/IgG1 ratio to a ratio of 1 (balanced Th1/Th2 response). **d**–**g** NAb responses. SARS-CoV-2-specific NAb (NT90 titer) induced by the vaccine vectors were measured after the first (**d**, **f**) and second (**e**, **g**) immunization against SARS-CoV-2 pseudovirus (pv) (**d**, **e**) or infectious SARS-CoV-2 virus (**f**, **g**) in pooled sera of immunized mice. Shown is the average NT90 measured in duplicate (**d**, **e**) or triplicate (**f**, **g**) infection. N/A = failed quality control of the samples. Dotted lines indicate lowest antibody dilution included in the analysis. **h** SARS-CoV-2/SARS-CoV-2pv correlation analysis. Correlation analysis of NT90 measured in mouse sera after one and two immunizations using infectious SARS-CoV-2 virus and SARS-CoV-2pv. Pearson correlation coefficient (*r*) was calculated in **h**; *0.05 < *p* ≪0.01, **0.01 < *p* < 0.001, ***0.001 < *p* < 0.0001, ****p < 0.0001; ns = not significant. Source data are provided as a Source Data file.

To determine the immunogenicity of the sMVA-vectored S and N antigens alone or combined, SARS-CoV-2-specific humoral and cellular immune responses were evaluated in Balb/c mice by two immunizations with the single and double recombinant vaccine vectors. High-titer antigen-specific binding antibodies were detected in all vaccine groups after the first immunization, and an increase in these responses was observed after the booster immunization (Figs. 5a, b and S3a, b). While the single recombinant vectors induced binding antibodies only against the S or N antigen, the double recombinant vectors induced binding antibodies against both the S and N antigens. In addition, all sMVA-CoV2 vectors encoding the S antigen (sMVA-S, sMVA-S/N, sMVA-N/S) stimulated high-titer binding antibodies against the S receptor binding domain (RBD), which is considered the primary target of NAb^22,24^. Antigen-specific binding antibody titers between the single and double recombinant vaccine groups were comparable. Notably, SARS-CoV-2 antigen-specific binding antibody responses stimulated by the sMVA-CoV2 vaccine vectors in mice exceeded SARS-CoV-2 S-, RBD-, and N-specific binding antibody responses measured in human convalescent immune sera (Figs. 5a, b and  S4). Analysis of the IgG2a/IgG1 isotype ratio of the binding antibodies revealed Th1-biased immune responses skewed toward IgG2a independently of the investigated vaccine group or antigen (Figs. 5c and S3c). Similar binding antibody responses to those induced by sMVA-CoV2 vectors in Balb/c mice were elicited by the vaccine vectors in C57BL/6 mice (Fig. S5).

Potent SARS-CoV-2-specific NAb responses as assayed using pseudovirus were detected after the first immunization in all vaccine groups receiving the vectors encoding the S antigen (sMVA-S, sMVA-S/N, sMVA-N/S), and these NAb responses increased after the booster immunization (Figs. 5d, e and S3d, e). Similar potent NAb responses as measured using pseudovirus were observed in the vaccine groups using infectious SARS-CoV-2 virus (Figs. 5f–h and S3f, g). We also evaluated the immune sera for potential of antibody-dependent enhancement of infection (ADE) using THP-1 monocytes. These cells do not express the ACE2 receptor, but express Fcγ receptor II, which is considered the predominant mediator of ADE in SARS-CoV infection^44^. THP-1 monocyte infection by SARS-CoV-2 pseudovirus was not promoted by immune sera of any of the vaccine groups even at sub-neutralizing antibody concentrations (Fig. S6), suggesting absence of Fc-mediated ADE by the vaccine-stimulated antibodies.

**Fig. 6:**
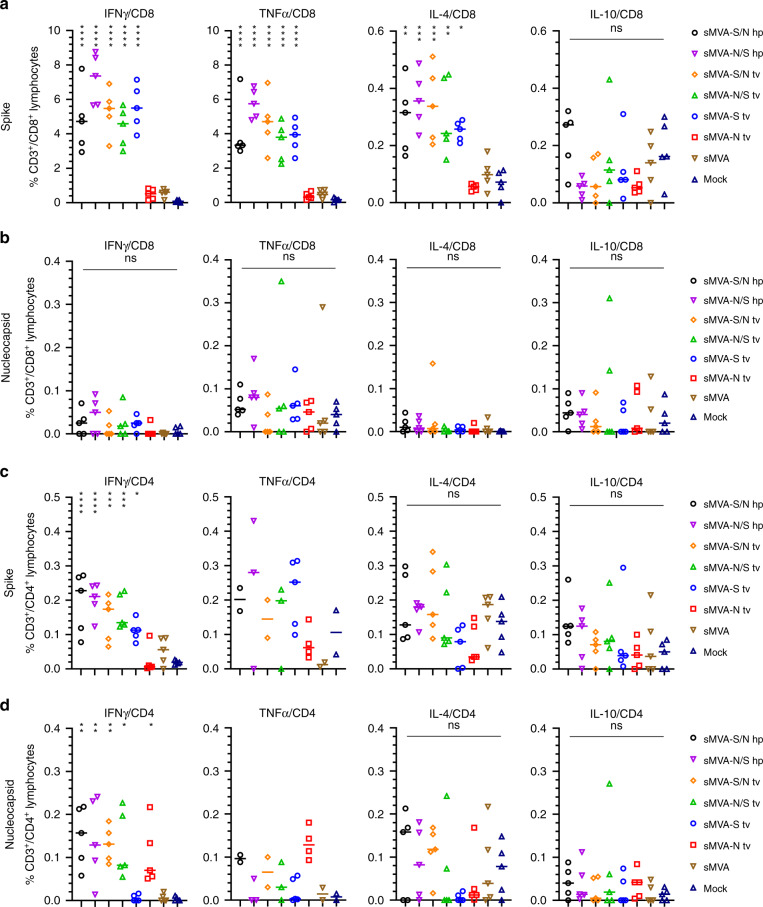
Cellular immune responses stimulated by sMVA-CoV2 vectors. Balb/c mice (*n* = 5) immunized twice in a 3-week interval with 5 × 10^7^ PFU of the single or double recombinant sMVA-CoV2 vectors derived with FPV HP1.441 (sMVA-S/N hp and sMVA-N/S hp) or TROVAC (sMVA-S/N tv, sMVA-N/S tv, sMVA-S tv, sMVA-N tv) were evaluated for SARS-CoV-2-specific cellular immune responses. Antigen-specific CD8+ (**a** and **b**) and CD4+ (**c** and **d**) T-cell responses induced by the vaccine vectors after two immunizations were evaluated by flow cytometry for IFNγ, TNFα, IL-4, and IL-10 secretion following ex vivo antigen stimulation using SARS-CoV-2 S- and N-specific peptide libraries. Due to technical issues, 1–3 animals/group were not included in the CD4/TNFα analysis in **c** and **d**. One-way ANOVA with Tukey’s multiple comparison test was used to compare % of cytokine-specific T cells between immunized mice and mock controls. Lines represent median values; *0.05 < *p* < 0.01, **0.01 < *p* < 0.001, ***0.001 < *p* < 0.0001, ****p < 0.0001; ns = not significant. Source data are provided as a Source Data file.

SARS-CoV-2-specific T cells evaluated after the second immunization by ex vivo antigen stimulation revealed both S- and N-specific T-cell responses in the vaccine groups receiving the double recombinant vectors sMVA-S/N and sMVA-N/S. In contrast, mice receiving the single recombinant vectors sMVA-N or sMVA-S developed T-cell responses only against either the N or S antigen (Fig. 6a–d and  S7 and S8). S-specific CD8+ T cells secreting IFNγ, TNFα, or IL-4 were measured in all vaccine groups receiving the S-encoding sMVA-CoV2 vectors, with particularly high IFNγ- and TNFα-secreting CD8+ T-cell levels that reached between 2% and 9% of the total CD8+ T-cell population (Fig. 6a). In addition, S-specific CD4+ T cells producing the Th1 cytokine IFNγ were measured at significant levels in all vaccine groups immunized with the S-encoding sMVA-CoV2 vectors, whereas no significantly elevated frequencies of S-specific CD4+ T cells producing Th2 cytokines (IL-4 and IL-10) were measured in these vaccine groups when compared to controls (Fig. 6c), indicating a Th1-biased response. While activated N-specific CD8+ T cells were not detected at significant frequency in any of the vaccine groups (Fig. 6b), N-specific IFNγ-secreting CD4+ T cells were measured in all animals vaccinated with the single or double recombinant vaccine vectors encoding N (Fig. 6d). Although TNFα secreting CD4+ T cells measured following N-specific peptide stimulation appeared elevated in all vaccine groups immunized with the single or double recombinant vectors encoding N, these T-cell responses were only of significant difference when comparing animals immunized with sMVA-N to the control groups (Figs. 6d and S8). No significant differences were observed in S- and N-specific T-cell levels for the single and double recombinant vaccine groups.

**Fig. 7:**
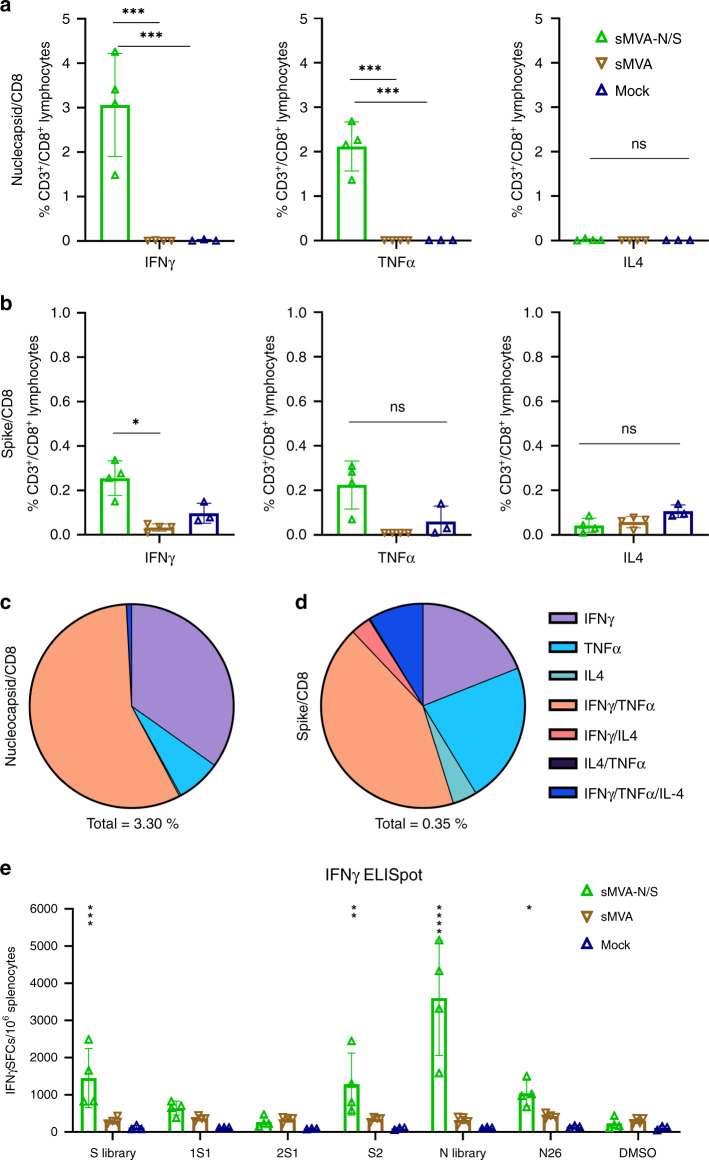
CD8+ T-cell responses induced by sMVA-N/S in HLA transgenic mice. HLA-B*07:02 (B7) transgenic mice (*n* = 4) were immunized twice in a 3-week interval with 1 × 10^7^ PFU of sMVA-N/S or sMVA control vector (reconstituted with FPV TROVAC). B7 mice (*n* = 3) were mock-immunized as additional control. Development of SARS-CoV-2-specific CD8+ T cells was evaluated 1 week post booster immunization. **a**–**d** Intracellular cytokine staining. Nucleocapsid- (**a**, **c**) and Spike-specific (**b**, **d**) CD8+ T cells were evaluated by intracellular cytokine staining for IFNγ, TNFα, and IL-4 secretion following ex vivo antigen stimulation by N and S peptide libraries, respectively. Panels **a** and **b** show the percentage of CD3+/CD8+ T cells secreting IFNγ, TNFα, or IL-4 following peptide stimulation. Panels **c** and **d** show relative frequencies of CD8+ T cells secreting one or more cytokines after peptide stimulation. Total percentage of cytokine-secreting cells within CD3+/CD8+ population is indicated under each pie chart. **e** ELISPOT analysis of IFNγ-secreting cells following stimulation with S and N peptide libraries, S library sub-pools (1S1, 2S1, S2), and N26 peptide containing the HLA-B*07:02-restricted N-specific immunodominant epitope SPRWYFYYL. One-way ANOVA with Dunnett’s multiple comparison test was used in **a** and **b**. Two-way ANOVA with Dunnett’s multiple comparison test was used in **e**. Data in **a**, **b**, and **e** are presented as mean values ± SD; *0.05 < *p* < 0.01, **0.01 < *p* < 0.001, ***0.001 < *p* < 0.0001, ****p < 0.0001; ns = not significant. Source data are provided as a Source Data file.

To further assess the immunogenicity of the sMVA-vectored N antigen, the double recombinant vaccine vector sMVA-N/S was evaluated for antigen-specific T-cell stimulation in transgenic C57BL/6 mice expressing the human leukocyte antigen (HLA)-B*0702 (B7). This HLA type has been recently described to present immunodominant N-specific peptides that are frequently recognized in SARS-CoV-2-infected patients^26,27^. C57BL/6 B7 mice immunized with sMVA-N/S developed high-frequency N-specific CD8+ T cells secreting IFNγ and TNFα that reached over 2–3% of the total CD8+ T-cell population (Fig. 7a). S-specific CD8+ cells secreting IFNγ were also detected at significant levels in sMVA-N/S-immunized C57BL/6 B7 mice, albeit at lower frequency compared to the N-specific T-cell responses (Fig. 7b). N- or S-specific CD8+ T cells secreting IL-4 were not observed in significant levels in sMVA-N/S-immunized animals. Notably, sMVA-N/S-stimulated CD8+ T cells to both the N and S antigens in C57BL/6 B7 mice were largely polyfuntional, with more than half of the N-specific CD8+ T cells secreting IFNγ and TNFα combined (Fig. 7c, d). Further analysis by IFNγ ELISPOT revealed that the S-specific T-cell responses induced by sMVA-N/S in C57BL/6 B7 mice were mostly directed toward epitopes of the S2 domain (Fig. 7e). In addition, a significant response was measured in sMVA-N/S-immunized B7 mice following stimulation with an HLA-B*0702 immunodominant N-specific peptide epitope (SPRWYFYYL) that has been shown recently to be recognized by a high proportion of people recovering from SARS-CoV2 disease (Fig. 7e)^27^. These results demonstrate that both the sMVA-vectored N and S antigens are immunogenic in HLA-B*0702 transgenic mice, while the CD8+ T-cell response targeting N appears to be highly dominant.

Stimulation of SARS-CoV-2-specific immune responses by both the S and N antigens was also evaluated in mice by co-immunization using the single recombinant vectors sMVA-S and sMVA-N at different doses. This study revealed similar SARS-CoV-2 antigen-specific humoral and cellular immune responses in vaccine groups receiving sMVA-S and sMVA-N alone or in combination (Figs. S9 and S10). Altogether these results indicate that the sMVA-vectored S and N antigens when expressed alone or combined using a single vector or two separate vectors can stimulate potent SARS-CoV-2-specific humoral and cellular immune responses in mice.

## Discussion

We developed a vaccine platform based on a fully synthetic form of the highly attenuated and widely used MVA vector. In response to the ongoing global SARS-CoV-2 pandemic, we used this vaccine platform to rapidly produce sMVA vectors co-expressing SARS-CoV-2 S and N antigens and show that these vectors can induce potent SARS-CoV-2 antigen-specific humoral and cellular immune responses in mice, including potent NAb. These results highlight the feasibility to efficiently produce recombinant MVA vectors from chemically synthesized DNA and to rapidly develop a synthetic poxvirus-based vaccine candidate to prevent SARS-CoV-2 infection. We envision that this vaccine platform based on synthetic DNA will facilitate the development of poxvirus vaccine vectors for infectious diseases of clinical importance and rapid response to new pandemics caused by emerging pathogens as in the case of SARS-CoV-2.

An elegant study by Noyce et al. recently described the construction of a synthetic horsepox virus from chemically synthesized DNA, but the utility of this approach to generate recombinant and immunogenic vaccine vectors was not demonstrated^45^. In contrast, we demonstrate the use of synthetic DNA to efficiently construct recombinant poxvirus-based vectors that enable potent immune stimulation to multiple heterologous antigens. When developing recombinant vaccine vectors based on MVA or other poxviruses, it is essential to evaluate different antigen forms and sequences, promoter elements, and insertion sites to optimize antigen expression, stability, and immunogenicity. In particular, the use of two or more insertion sites is a common strategy to efficiently and stably co-express multiple antigens inserted into MVA^10,41,43^. Through advances in synthetic biology, we were able to construct a unique and simplified three-plasmid system that allows to simultaneously insert two or more antigen sequences into different commonly used MVA insertion sites (Del2, Del3, and IGR69/70) and to concomitantly combine these antigen sequences in a single recombinant MVA vector. This approach to rapidly combine multiple antigens in a single MVA vector would be difficult to achieve by conventional methods based on in vitro recombination or BAC technology^1,3,34^, which rely on labor-intensive and successive antigen insertion steps when utilizing different genome positions. While we have demonstrated the potential of this vaccine platform to rapidly generate multi-antigenic SARS-CoV-2 vaccines, this technology could be easily applied to rapidly develop virtually any kind of multi-antigenic vaccine vector for many types of disease indications. Moreover, this technology could form the basis to synthetically design optimized vaccine platforms based on MVA or other poxviruses to generate recombinant vaccines vectors tailored to specific diseases or patient-specific needs.

In contrast to the approach by Noyce et al. to produce a synthetic horsepox virus that involves the use of multiple smaller linear DNA fragments (~10–30 kbp) and chemical ligation of terminal HL sequences^45^, we demonstrate efficient sMVA virus reconstitution using circular forms of three large synthetic ∼60 kbp DNA fragments with embedded HL and CR sequences. The use of circular plasmids for the sMVA reconstitution is a major advantage over the approach by Noyce et al. as it allows easy maintenance and manipulation of the sMVA fragments in *E. coli* and to generate single, double, or triple recombinant sMVA vectors by simple co-transfection of the three plasmids into helper virus-infected cells without the need for additional purification steps or modifications (Fig. 4). We hypothesize that the intrinsically added CR/HL/CR sequence elements are essential for the sMVA reconstitution using circular DNA to promote the resolution of the sMVA fragments from the plasmid backbones and subsequent recombination and packaging as full-length genomes^28–33^. Whether the CR/HL/CR sequence are needed at both ends of all three synthetic fragments to promote sMVA virus reconstitution or whether they are only needed at the genomic termini of the ITRs in sMVA fragments F1 and F3 requires further investigation (Fig. 1). Although the precise mechanism of the virus reconstitution by the synthetic vaccine platform was not investigated, we demonstrate that the three circular DNA fragments efficiently recombine with one another and enable rapid generation of functional and highly immunogenic sMVA vaccine vectors expressing multiple heterologous antigens.

In contrast to most other currently employed SARS-CoV-2 vaccine approaches that solely rely on the S antigen, our SARS-CoV-2 vaccine approach using sMVA employs immune stimulation by S and N antigens, which both are implicated in protective immunity^20,26^. While the S protein is essential for the stimulation of protective NAb, the N protein is abundantly expressed, highly immunogenic, and has the advantage of being more conserved than the S protein and could therefore contribute to the stimulation of broadly effective immune responses^46^. The observation that the sMVA-CoV2 vectors co-expressing S and N antigens can stimulate potent NAb against SARS-CoV-2 pseudovirus and infectious virions suggests that they can elicit antibodies that are considered effective in preventing SARS-CoV-2 infection and disease^16,18,20,21^. In addition, we demonstrate that these vectors have the capacity to stimulate antibody responses to both the S and N antigens in Balb/c and C57BL/6 mice that exceed humoral responses measurable in human convalescent immune sera. This includes potent antibody responses to the RBD, which is thought to be the primary target of NAb during SARS-CoV-2 infection, further highlighting the immunogenicity of these vectors to elicit potentially protective immune responses. We show that the vaccine vectors stimulate a Th1-biased antibody and cellular immune response, which is considered the preferred antiviral adaptive immune response to avoid vaccine-associated enhanced respiratory disease^47,48^. We did not find any evidence for Fc-mediated ADE promoted by the vaccine-induced immune sera, suggesting that the antibody responses induced by the vaccine vectors bear minimal risk for ADE-mediated immunopathology, a general concern in SARS-CoV-2 vaccine development^47,48^. In addition, based on findings with other viruses associated with ADE, the stimulation of Th1 immunity with a strong T-cell response component appears to be the way forward to develop an effective SARS-CoV-2 vaccine candidate^49^.

Immune responses besides NAb targeting the S antigen may contribute to protection against SARS-CoV-2 infection, which is highlighted by the finding that even patients without measurable NAb can recover from SARS-CoV-2 infection^20^ and by correlation of lymphopenia with disease severity^50^. While antibodies could be particularly important to prevent initial SARS-CoV-2 acquisition, T-cell responses may add an additional countermeasure to control sporadic virus spread at local sites of viral infection, thereby limiting virus transmission. In addition, based on observations with SARS-CoV-1, T-cell responses to SARS-CoV-2 are believed to be more durable than NAb responses^51^ and are most likely essential for the stimulation of long-lasting protective immunity^52^. The capacity of the double recombinant sMVA-CoV-2 vectors to induce high-frequency S-specific CD8+ T cells as well as CD4+ T cells to both the S and N antigens in Balb/c mice seems consistent with the magnitude and phenotype of cellular immune responses in SARS-CoV-2-infected patients. Moreover, the potent immunogenicity of these vaccine vectors (exemplified by sMVA-S/N) in HLA-B*07:02 transgenic mice to stimulate a highly dominant CD8+ T-cell response of polyfunctional phenotype toward the N antigen suggests that they have the capacity to stimulate HLA-restricted cellular immune responses similar to those frequently detected in individuals recovering from SARS-CoV-2 disease^26,27^. These findings further validate the incorporation of both the S and N antigens into an sMVA-based vaccine candidate to elicit robust and broadly effective T-cell responses against SARS-CoV-2.

Our dual recombinant vaccine approach based on sMVA to induce robust humoral and cellular immune responses to S and N antigens may provide protection against SARS-CoV-2 infection beyond other vaccine approaches that solely employ the S antigen. Evaluation of the protective efficacy of the double recombinant sMVA-CoV2 vaccine vectors in animal models is the next step toward the goal of advancing one of these vaccine candidates into clinical evaluation.

## Methods

### Cells and viruses

BHK-21 (CCL-10), A549 (CCL-185), HeLa (CCL-2), 293T (CRL-1573), 143B (CRL-8303), HEK293/17 (CRL11268), THP-1 (TIB-202), and ARPE-19 (CRL-2302) were purchased from the American Type Culture Collection (ATCC), and grown according to ATCC recommendations. CEFs were purchased from Charles River (10100795) and grown in minimum essential medium (MEM) with 10% FBS (MEM10). HEK293T/ACE2 were a kind gift of Pamela J. Bjorkman^53^. We acknowledge Bernard Moss (LVD, NIAID, NIH) for the gift of wtMVA (NIH Clone 1) that was used solely as a reference standard. To produce sMVA and wtMVA virus stocks, CEFs were seeded in 30 × 150 mm tissue culture dishes, grown to ~70–90% confluency, and infected at 0.02 multiplicity of infection (MOI) with sMVA, wtMVA, or sMVA-CoV2 vectors. Two days post infection, ultra-purified virus was prepared by 36% sucrose cushion ultracentrifugation and virus resuspension in 1 mM Tris-HCl (pH 9)^54^. Virus stocks were stored at −80 °C. Virus titers were determined on CEF by immunostaining of viral plaques at 16–24 h post infection using polyclonal Vaccinia antibody (9503-2057, Bio-Rad. Dilution 1:2000). FPV stocks were produced following propagation on CEF using FPV strain TROVAC (ATCC VR-2553)^36^ or HP1.441 (ref. ^37^), kindly provided by Bernard Moss. FPV titers were evaluated on CEF by virus plaque determination. SARS-CoV-2 strain USA-WA1/2020 (BEI Resources NR-52281) was used in the focus reduction neutralization test (FRNT) assay^55^.

### Construction of sMVA fragments

The three ~60 kbp sMVA fragments (F1–F3; Fig. 1) comprising the complete MVA genome sequence reported by Antoine et al. (NCBI Accession# U94848)^4^ were constructed as follows: sMVA F1 contained base pairs 191-59743 of the MVA genome sequence; sMVA F2 contained base pairs 56744-119298 of the MVA sequence; and sMVA F3 contained base pairs 116299-177898 of the reported MVA genome sequence^4^. A CR/HL/CR sequence arrangement composed of 5′-TTT TTT TCT AGA CAC TAA ATA AAT A*GT AAG ATT AAA TTA ATT ATA AAA TTA TGT ATA TAA TAT TAA TTA TAA AAT TAT GTA TAT GAT TTA CTA ACT TTA GTT AGA TAA ATT AAT AAT ACA TAA ATT TTA GTA TAT TAA TAT TAT AAA TTA ATA ATA CAT AAA TTT TAG TAT ATT AAT ATT ATA TTT TAA A*TA TTT ATT TAG TGT CTA GAA AAA AA-3′ was added in the same orientation to both ends of each of the sMVA fragments, wherein the italicized letters indicate the duplex copy of the MVA terminal HL sequence and the underlined letters indicate the CR sequences. Notably, the CR/HL/CR sequences incorporated at the ITRs of sMVA F1 and F3 were added in identical arrangement as the CR/HL/CR sequences occur at the ITRs at the genomic junctions of putative MVA replication intermediates^4^. The sMVA fragments were produced and assembled by Genscript using chemical synthesis, combined with a yeast recombination system. All sMVA fragments were cloned into a yeast shuttle vector, termed pCCI-Brick, which contains a mini-F replicon for stable propagation of large DNA fragments as low copy BACs in *E. coli*. sMVA F1 and F3 were cloned and maintained in EPI300 *E. coli* (Epicentre), while sMVA F2 was cloned and maintained in DH10B *E. coli* (Invitrogen). The synthetic fragments and the vaccine constructs derived thereof that are described in this study are available from the corresponding authors upon request under a material transfer agreement.

### Antigen insertion

SARS-CoV-2 S and N antigen sequences were inserted into the sMVA fragments by En passant mutagenesis in GS1783 *E. coli* cells^39,40^. Using the pGEM-T-mH5 vector that we have previously generated^9^, transfer constructs were generated that consisted of the S or N antigen gene sequence with upstream mH5 promoter sequence and downstream Vaccinia transcription termination signal (TTTTTAT). Additionally, a kanamycin resistance cassette with adjacent I-SceI homing endonuclease restriction site and a flanking 50-bp gene duplication was introduced into the S and N antigen sequences^8^. S and N antigen sequences were based on the SARS-CoV-2 reference strain (NCBI Accession# NC_045512) and codon-optimized for Vaccinia^10^. Codon-optimized S and N gene sequences were synthesized by Twist Biosciences. The transfer constructs were amplified by PCR with Phusion polymerase (Thermo Fisher Scientific) using primers providing ~50 bp extensions for homologous recombination to insert the transfer constructs into the sMVA fragments by Red-recombination^39,40^. Primers 5′-AAA AAA TAT ATT ATT TTT ATG TTA TTT TGT TAA AAA TAA TCA TCG AAT ACG AAC TAG TAT AAA AAG GCG CGC C-3′ and 5′-GAA GAT ACC AAA ATA GTA AAG ATT TTG CTA TTC AGT GGA CTG GAT GAT TCA AAA ATT GAA AAT AAA TAC AAA GGT TC-3′ were used to insert the N antigen sequence into the Del2 site. Primers 5′-ATA TGA ATA TGA TTT CAG ATA CTA TAT TTG TTC CTG TAG ATA ATA ACT AAA AAT TTT TAT CTA GTA TAA AAA GGC GCG CC-3′ and 5′-GGA AAA TTT TTC ATC TCT AAA AAA AGA TGT GGT CAT TAG AGT TTG ATT TTT ATA AAA ATT GAA AAT AAA TAC AAA GGT TC-3′ were used to insert the S antigen sequence into the IGR69/70 insertion site. Primers 5′-TTG GGG AAA TAT GAA CCT GAC ATG ATT AAG ATT GCT CTT TCG GTG GCT GGT AAA AAA TTG AAA ATA AAT ACA AAG GTT C-3′ and 5′-ACA AAA TTA TGT ATT TTG TTC TAT CAA CTA CCT ATA AAA CTT TCC AAA TAC TAG TAT AAA AAG GCG CGC C-3′ were used to insert the S or N antigen sequence into the Del3 site. Underlined letters indicate sequences used to produce ~50 bp extensions for homologous recombination. The amplified PCR products were purified using the NucleoSpin Gel and PCR clean-up kit (Macherey–Nagel) and 100 ng of PCR product was electroporated at 15 kV/cm, 25 µF, and 200 Ω into 50 µL of recombination-competent GS1783 bacteria harboring the sMVA fragments. The bacteria were re-suspended in 1 mL of Luria-Bertani (LB) medium without antibiotics and incubated for 2 h at 32 °C and 220 r.p.m. After 2 h of incubation, the bacteria were streaked onto LB agar plates with 30 µg/mL chloramphenicol and 30 µg/mL kanamycin and incubated at 32 °C for 2 days. Bacterial clones harboring sMVA fragments with inserted antigen sequences at the respective MVA insertion sites were identified by PCR and restriction pattern analysis. To seamlessly remove the kanamycin resistance marker from the inserted antigen sequences by a I-SceI-mediated second Red-recombination reaction, 100 µL of an overnight culture of selected bacterial clones was added to 900 µL of LB medium containing 30 µg/mL chloramphenicol and incubated for 1.5–2 h at 32 °C and 220 r.p.m. Subsequently, 1 mL of LB containing 30 µg/mL chloramphenicol and 2% l-arabinose was added to induce the expression of the I-SceI homing endonuclease enzyme and to induce a double-strand break at the 50 bp gene duplication. The bacteria were incubated for 1 h at 32 °C and then transferred to a water bath and incubated for 30 min at 220 r.p.m. and 42 °C to induce the expression of the Red-recombination proteins and to mediate the removal of the kanamycin resistance marker by recombination of the 50 bp gene duplication. After an additional incubation period of the bacteria for 2 h at 32 °C and 220 r.p.m., the bacteria were streaked onto LB agar plates with 30 µg/mL chloramphenicol and 1% l-arabinose and incubated at 32 °C for 2 days. Bacterial clones harboring sMVA fragments with seamlessly removed kanamycin marker from the inserted antigen sequences were identified by PCR, restriction pattern analysis, and Sanger sequencing.

### sMVA virus reconstitution

sMVA virus reconstitution from the three sMVA plasmids in BHK cells using FPV as a helper virus was performed as follows^8–10^. The three sMVA plasmids were isolated from *E. coli* by alkaline lysis^56^ and co-transfected into 60–70% confluent BHK cells grown in 6-well tissue culture plates using Fugene HD transfection reagent (Roche) according to the manufacturer’s instructions. At 4 h post transfection, the cells were infected with ~0.1–1 MOI of FPV to initiate sMVA virus reconstitution. The transfected/infected BHK cells were grown for 2 days and then every other day re-seeded in ~1:2 ratio and grown for additional 2 days in larger tissue culture formats over a period of 8–12 days until most or all of the cells showed signs of sMVA virus infection. Using this procedure, characteristic MVA viral plaque formation and cytopathic effects (CPEs) indicating sMVA virus reconstitution were usually detected at 4–8 days post transfection/infection. Fully infected BHK cell monolayers were usually visible at 8–12 days post transfection/infection. sMVA virus from infected BHK cell monolayers was prepared by conventional freeze/thaw method and passaged once on BHK cells before producing ultra-purified sMVA virus stocks using CEF. sMVA vectors or recombinant sMVA-CoV2 vaccine vectors were reconstituted either with FPV strain HP1.441 or with FPV strain TROVAC.

### Host cell range

Comparison of sMVA and wtMVA host cell range using various human cell lines (HeLa, 293T, A549, and 143B), BHK cells, and CEF was performed as follows. The cells were seeded in 6-well-plate tissue culture format and at 70–90% confluency infected in duplicates at 0.01 MOI with sMVA or wtMVA using MEM with 2% FBS (MEM2). At 2 h post infection, the cells were washed twice with PBS and incubated in normal growth medium for 2 days. After the incubation period, the virus was prepared by conventional freeze/thaw method and the virus titers of each duplicate infection were determined in duplicates on CEF.

### Replication kinetics

To compare the replication kinetics of sMVA and wtMVA, CEF or BHK cells were seeded in 6-well-plate tissue culture format and at 70–90% confluency infected in triplicates at 0.02 MOI with sMVA or wtMVA using MEM2. After 2 h of incubation, the cells were grown in MEM10. At 24 and 48 h post infection, the virus was prepared by freeze/thaw method and the virus titers of each triplicate infection and the inoculum were determined on CEFs.

### Plaque size analysis

To compare the foci size of sMVA and wtMVA, CEF or BHK cells were seeded in 6-well-plate tissue culture format and at 70–90% confluency infected with 0.002 MOI of sMVA or wtMVA using MEM2. After 2 h of incubation, MEM10 was added and the cells were grown for 16–24 h. The cell monolayers were stained with Vaccinia virus polyclonal antibody and viral foci were imaged using Leica DMi8 inverted microscope and measured using LAS X software (v2.0.0.14332). The size of 20 viral foci per sMVA or wtMVA was calculated using the formula Area = *π* × *a* × *b*, where *a* and *b* are the major and minor radii of the ellipse, respectively.

### PCR analysis

To characterize the viral DNA of the sMVA vectors by PCR, CEFs were seeded in 6-well-plate tissue culture format and at 70–90% confluency infected at 5 MOI with sMVA vectors or wtMVA. DNA was extracted at 16–24 h post infection by the DNA Easy Blood and Tissue Kit (Qiagen) according to the manufacturer’s instructions. All PCR reactions were performed with Phusion polymerase (Thermo Fisher Scientific). Primers 5′-TCG TGG TGT GCC TGA ATC G-3′ and 5′-AGG TAG CGA CTT CAG GTT TCT T-3′ were used to detect MVA ITR sequences; primers 5′-TAT CCA CCA ATC CGA GAC CA-3′ and 5′-CCT CTG GAC CGC ATA ATC TG-3′ were used to verify the transition from the left ITR into the unique region; primers 5′-AGG TTT GAT CGT TGT CAT TTC TCC-3′ and 5′-AGA GGG ATA TTA AGT CGA TAG CCG-3′ were used to verify the Del2 site with or without inserted N antigen sequence; primers 5′-TGG AAT GCG TTC CTT GTG C-3′ and 5′-CGT TTT TCC CAT TCG ATA CAG-3′ with binding sites flanking the F1/F2 homologous sequences were used to verify the F1/F2 recombination site; primers 5′-TAT AGT CTT TGT GGC ATC CGT TG-3′ and 5′-ACC CAA ACT TTA GTA AGG CCA TG-3′ were used to verify the IGR69/70 insertion site with or without inserted S antigen; primers 5′-ATA AGC GTT GTC AAA GCG GG-3′ and 5′-AGG AAA TAG AAA TTG TTG GTG CG-3′ with binding sites flanking the F2/F3 homologous sequences were used to verify the F2/F3 recombination site; primers 5′-ACA TTG GCG GAC AAT CTA AAA AC-3′ and 5′-ATC ATC GGT GGT TGA TTT AGT AGT G-3′ were used to verify the Del3 insertion site with and without inserted S or N antigen sequences; primers 5′-TAT CCA CCA ATC CGA GAC CA-3′ and 5′-GTC TGT CCG TCT TCT CTA TTG TTT A-3′ were used to verify the transition from the unique region into the right ITR; and primers 5′-TTA ACT CAG TTT CAA TAC GGT GCA G-3′ and 5′-TGG GGT TTC TTC TCA GGC TAT C-3′ were used to detect the *SopA* element of the BAC vector. PCR products were analyzed by agarose gel electrophoresis and imaged using Syngene PXi6 imager with GeneSys (v1.5.4.0) software. Uncropped gel images are provided as Source Data file. To sequence the PCR products derived from the sMVA vectors, the amplified PCR products were purified using the NucleoSpin Gel and PCR Clean-up Kit (Macherey–Nagel) according to the manufacturer’s instructions and analyzed by Sanger sequencing.

### Restriction pattern analysis

BHK cells were seeded in 20 × 150 mm tissue culture dishes, grown to ~70–90% confluency, and infected at 0.01 MOI with wtMVA, sMVA tv1, or sMVA tv2. Ultra-purified virus was prepared 2 days post-infection by 36% sucrose cushion ultracentrifugation and virus resuspension in 1 mM Tris-HCl (pH 9)^54^. Viral DNA was phenol/chloroform extracted, followed by ethanol precipitation^57^. Briefly, isolated virus particles were resuspended in lysis buffer (50 mM Tris-HCl pH 8.0, 1.2% SDS, 4 mM EDTA pH 8.0, 4 mM CaCl_2_, and 0.4 mg/mL proteinase K) and incubated overnight at 37 °C. DNA was extracted twice with phenol; each extraction was performed by adding an equal volume of buffered phenol and centrifugation at room temperature (RT) for 10 min at 300 × *g*. Aqueous phase was carefully collected to avoid DNA shearing. Final extraction was performed by adding equal volume of 1:1 phenol/chloroform to aqueous phase, followed by centrifugation as described above, and completed by ethanol precipitation of phenol/chloroform extracted viral DNA. DNA concentration and *A*_260_/*A*_280_ ratios were determined using NanoVue (GE Healthcare Bio-sciences Corp.); 10 μg of viral DNA was digested with 3 units of either KpnI or XhoI, followed by visualization on 0.5% EtBr-stained agarose gel that was run at 2.4 v/cm, overnight. Images were acquired using Syngene PXi6 imager with GeneSys (v1.5.4.0) software.

### Sequencing of sMVA fragments and sMVA vectors

PacBio (Pacific Biosciences) Long Read Sequencing analysis was used to determine the sequences of the cloned sMVA fragments (F1–F3) and reconstituted sMVA vectors. Plasmid DNA for sequencing the sMVA fragments was isolated by QIAGEN Large-Construct Kit according to the manufacturer’s instructions. Viral DNA for sequencing sMVA was isolated from purified virus particles by phenol/chlorophorm extraction as described above^57^. Viral DNA for sequencing the sMVA-CoV2 vectors was isolated from purified virus particles by NucleoSpin Blood QuickPure DNA extraction kit (Macherey–Nagel) according the manufacturer’s instructions. Briefly, 5 μg of fragmented DNA was converted to barcoded SMRTbell libraries using the SMRTbell Template Prep Kit 1.0 and Barcoded Adapter Plate-96 (PacBio). Libraries of the sMVA fragments and sMVA vector were size-selected (7-kb size cutoff) with BluePippin (Sage Science). After polymerase binding to the libraries with sequencing primers, the polymerase complexes were loaded into RSII SMRT cells using MagBeads loading and sequenced on PacBio RSII with 6 h movie. The polymerase complexes of sMVA-CoV2 vectors were loaded into a Sequel SMRT cell using diffusion mode and sequenced on PacBio Sequel with 10 h movie. Read demultiplexing, read mapping to the reference sequences, and Circular Consensus Sequencing (CCS) analyses were performed by Demultiplex Barcodes, Resequencing, and CCS modules, respectively, either in SMRT Portal (v. 2.3.0) or SMRT Link (v6.0.0.47841) or SMRT Link (v8.0.0.80529). The variants calling with CCS reads were carried out using VarScan v2.3.9 after mapping CCS reads using pbmm2v 1.0.0. De novo assembly was done using canu v1.7.1. The 5′ start position of the assembled contig was edited by comparing to the references. MVA U94848.1 was used as a reference for mapping the reads of the sMVA genome sequence. Sequences of the sMVA fragments and sMVA-CoV2 vectors were mapped via alignment with corresponding reference sequences based on MVA U94848.1 that were constructed by Vector NTI (Invitrogen, v. 11.5). Along with the comparison of de novo assembled contig to each reference, this analysis confirmed the sequence identity of the cloned sMVA fragments and reconstituted sMVA vectors, including a single point mutation in a non-coding determining region at 3 base pairs downstream of 021L^4^ that was found in sMVA fragment F1 and all sequenced reconstituted sMVA vectors (sMVA and sMVA-CoV2 vectors). An additional variation (point mutation) that could not be unambiguously excluded was found in a non-coding determining region at the tandem repeats 88 bp from the end of the ITR within sMVA fragment F3. As these two variations were present in the cloned sMVA fragments, they were confirmed as errors originating during the chemical synthesis of the sMVA fragments. The internal unique region and unique regions of the ITRs encompassing the complete MVA coding content could be reliably assembled for all reconstituted sMVA vectors. The sequence contig of the sMVA vector covered almost (over 99%) the complete U94848.1 reference sequence, with only a few exceptions at the highly repetitive ITR tandem repeats. The complete regions of the ITR tandem repeats of the sMVA-CoV2 vectors could not be reliably mapped through alignment with the reference sequences or de novo assembly due to low coverage at these regions, likely as a result of the quality of the sequence reads. Reference sequences of the sMVA fragments and sMVA-CoV2 vectors based on the PacBio sequencing were deposited in NCBI. To determine the absence of contaminating BAC vector sequences in the raw sequencing data of the reconstituted sMVA vectors, the sequencing reads were aligned onto the reference pCCl-Brick vector sequence provided by Genscript using the resequencing module in SMRT Link (v8.0.0.80529).

### Immunoblot analysis

BHK cells infected at 5 MOI were harvested 24 h post infection. Proteins were solubilized in PBS with 0.1% Triton X-100, supplemented with protease inhibitor, then reduced and denatured in Laemmli buffer containing DTT and boiled at 95 °C for ~10 min. Proteins were resolved on a 4–20% Mini Protean TGX gradient gel (BioRad) and transferred onto PVDF membrane. S protein was probed with anti-SARS-CoV-1 S1 subunit rabbit polyclonal antibody at a dilution of 1:1300 (40150-T62-COV2, Sino Biological); N protein was probed with anti-SARS-CoV1 NP rabbit polyclonal antibody (40413-T62, Sino Biological) was used at dilution of 1:10,000. Vaccinia B5R protein was probed as a loading control using 19C2 (ref. ^58^) rat monoclonal antibody (hybridoma supernatant) at a dilution of 1:20. Anti-rabbit IgG polyclonal antibody and anti-rat IgG polyclonal antibody conjugated with horseradish peroxidase (A6154 and A5795, Sigma-Aldrich) were used as a secondary antibody at a dilution of 1:5000 and 1:3300, respectively. Protein bands were visualized with a chemiluminescent substrate (Thermo Fisher Scientific).

### Flow cytometry

HeLa cells were seeded in a 6-well plate (5 × 10^5^/well) and infected the following day with sMVA vaccine candidates at an MOI of 5. Following an incubation of 6 h, cells were detached with non-enzymatic cell dissociation buffer (13151014, GIBCO). Cells were either incubated directly with primary antibody or fixed and permeabilized prior to antibody addition. Anti-SARS-CoV-1 S1 rabbit (40150-R007, Sino Biological) and S2 mouse (GTX632604, GeneTex) monoclonal antibodies, anti-SARS-CoV-1 N rabbit monoclonal antibody (40143-R001, Sino Biological), and anti-Vaccinia rabbit polyclonal antibody (9503-2057, Bio Rad) were used in dilution 1:2000. One hour later, anti-mouse or anti-rabbit Alexa Fluor 488-conjugated secondary antibodies (A11001, A21206; Invitrogen) were added to the cells at a dilution of 1:4000. For double staining with S2- and N-specific antibodies, anti-mouse Alexa Fluor 488 and anti-rabbit Alexa Fluor 647-conjugated secondary antibodies (A21244; Invitrogen) were used. Live cells were ultimately fixed with 1% paraformaldehyde (PFA) and acquired using a BD FACSCelesta flow cytometer with BD FACSDiva software (v8.0.1.1). Analysis was performed using FlowJo (v10.6.2).

### Immunofluorescence

BHK or HeLa cells were grown on glass coverslips and infected with sMVA or recombinant sMVA encoding S and/or N antigens at an MOI of 5 for 6 h at 37 °C in a humidified incubator (5% CO_2_). After infection, cells were fixed for 15 min in 2% PFA and then directly permeabilized by addition of ice cold 1:1 acetone/methanol for 5 min on ice. Cells were blocked for 1 h with 3% BSA at RT, incubated with primary antibody mix (1:500) against the S2 subunit or N for 1 h at 37 °C, and then incubated with Alexa-conjugated secondary antibodies (Thermo Fisher) (1:2000) for 1 h at 37 °C, with washing (PBS + 0.1% Tween20) between each step. For detection of cell membranes and nuclei, cells were incubated with Alexa-conjugated wheat germ agglutinin at 5 μg/mL (W11263, Thermo Fisher Scientific) and DAPI for 10 min at RT. Coverslips were washed and mounted onto slides with Fluoromount-G (SouthernBiotech). Microscopic analysis was performed using the confocal microscope LSM700 (Zeiss). Images were acquired and processed using Zen software (Zeiss, Black Edition Version 8.1).

### Mouse immunization

The Institutional Animal Care and Use Committee (IACUC) of the Beckman Research Institute of City of Hope (COH) approved protocol 20013 assigned for this study. All study procedures were carried out in strict accordance with the recommendations in the Guide for the Care and Use of Laboratory Animals and the Public Health Service Policy on the Humane Care and Use of Laboratory Animals. Mice were kept on a 12-h light/12-h dark cycle, at 22–24 °C and 30–70% humidity, with ad libitum access to food and water. Six-week-old C57BL/6 (C57BL/6J, 000664) or Balb/c (BALB/cJ, 000651) mice were purchased from the Jackson laboratories. C57BL/6 Nramp were bred at the City of Hope animal facility. HLA-B*0702 H-2KbDb double-knockout (B7) transgenic mice on a C57BL/6 background were obtained from F. Lemonnier^59^ (Institut Pasteur, France) and bred at the City of Hope Animal Research Center. Mice (*N* = 4–5) were immunized twice in 3 weeks interval by intraperitoneal route with 5 × 10^7^ PFU (high dose) or 1 × 10^7^ PFU (low dose) of sMVA, wtMVA, or sMVA-CoV2 vectors. B7 mice were immunized with 1 × 10^7^ PFU of sMVA-N/S or sMVA control vector. To determine immune stimulation by both the S and N antigens when using separate vectors (Figs. S9 and S10), mice were co-immunized via the same immunization schedule and route with half of the high (2.5 × 10^7^ PFU) or low dose (0.5 × 10^7^ PFU) of each of the vaccine vectors. Blood samples for humoral immune analysis were collected by retro-orbital bleeding 2 weeks post-prime and 1-week post booster immunization. Splenocytes for cellular immune analysis were collected at 1 week post booster immunization and were isolated by standard procedure after animals were humanely euthanized.

### Binding antibodies

Binding antibodies in mice immunized with sMVA, wtMVA, or sMVA-CoV2 vectors were evaluated by ELISA. ELISA plates (3361, Corning) were coated overnight with 1 µg/mL of MVA expressing Venus fluorescent marker^9^, S (S1 + S2, 40589-V08B1, Sino Biological), RBD (40592-V08H, Sino Biological), or N (40588-V08B, Sino Biological). Plates were blocked with 3% BSA in PBS for 2 h. Serial dilutions of the mouse sera were prepared in PBS and added to the plates for 2 h. After washing the plate, 1:3000 dilution of HRP-conjugated anti-mouse IgG secondary antibody (W402B, Promega) was added and incubated for one additional hour. Plates were developed using 1-Step Ultra TMB-ELISA (34028, Thermo Fisher) for 1–2 min after which the reaction was stopped with 1 M H_2_SO_4_. Plates were read at 450 nm wave length using FilterMax F3 microplate reader and SoftMax Pro software v7 (Molecular Devices). Binding antibodies endpoint titers were calculated as the latest serum dilution to have an absorbance higher than 0.1 absorbance units (OD) or higher than the average OD in mock immunized mice plus five times the standard deviation of the OD in the same group at the same dilution. For evaluation of the IgG2a/IgG1 and IgG2c/IgG1 ratios, mouse sera were diluted 1:10,000 in PBS. The assay was performed as described above except for the secondary antibodies (1:2000. Goat anti-mouse IgG2a cross absorbed HRP antibody, Southern biotech, 1083-05; Goat anti-mouse IgG1 cross absorbed HRP antibody, Thermo Fisher, A10551; Goat anti-mouse IgG2c HRP antibody, Thermo Fisher, PA1-29288). The IgG2a/IgG1 and IgG2c/IgG1 ratios were calculated by dividing the absorbance read in the well incubated with the IgG2a/IgG2c secondary antibody by the absorbance for the same sample incubated with the IgG1 antibody.

### MVA neutralization assay

ARPE-19 cells were seeded in 96-well plates (1.5 × 10^4^ cells/well). The following day, serial dilutions of mouse sera were incubated for 2 h with MVA expressing the fluorescent marker Venus^9^ (1.5 × 10^4^ PFU/well). The serum–virus mixture was added to the cells in duplicate wells and incubated for 24 h. After the 24 h incubation period, the cells were imaged using Leica DMi8 inverted microscope. Pictures from each well were processed using Image-Pro Premier (v9.2; Media Cybernetics) and the fluorescent area corresponding to the area covered by MVA-Venus infected cells was calculated.

### SARS-CoV-2 pseudovirus production

The day before transfection, HEK293T/17 were seeded in a 15-cm dish at a density of 5 × 10^6^ cells in DMEM supplemented with 10% heat inactivated FBS, non-essential amino acids, HEPES, and glutamine^60^. Next day, cells were transfected with a mix of packaging vector (pALDI-Lenti System, Aldevron), luciferase reporter vector, and a plasmid encoding for the wild-type SARS-CoV2 Spike protein (VG40589-UT, Sino Biological) or vesicular stomatitis virus G (VSV-G, Aldevron), using FuGENE6 (Roche) as a transfection reagent:DNA ratio of 3:1, according to manufacturer’s protocol. Sixteen hours post-transfection, the media was replaced and cells were incubated for an additional 24–72 h. Supernatants were harvested at 24, 48, and 72 h, clarified by centrifugation at 1500 r.p.m. for 5 min and filtered using a sterile 0.22-µm pore size filter. Clarified lentiviral particles were concentrated by ultracentrifugation at 20,000 r.p.m. for 2 h at 4 °C. The pellet was resuspended in DMEM containing 2% heat-inactivated FBS and stored overnight at 4 °C to allow the pellet to completely dissolve. Next day, the samples were aliquoted, snap frozen, and stored at −80 °C for downstream assays.

### SARS-CoV-2 pseudotype neutralization and ADE assay

Levels of p24 antigen in the purified SARS-CoV-2 pseudotype solution were measured by ELISA (Takara). Mouse sera were heat inactivated, pooled and diluted at a linear range of 1:100 to 1:50,000 in complete DMEM. For the neutralization assay, diluted serum samples were pre-incubated overnight at 4 °C with SARS-CoV-2-Spike pseudotyped luciferase lentiviral vector, normalized to 100 ng/mL of p24 antigen. HEK293T cells overexpressing ACE-2 receptor were seeded the day before transduction at a density of 2 × 10^5^ cells per well in a 96-well plate in complete DMEM^53^. Before infection, 5 µg/mL of polybrene was added to each well. Neutralized serum samples were then added to the wells and the cells were incubated for an additional 48 h at 37 °C and 5% CO_2_ atmosphere. Following incubation, cells were lysed using 40 µL of Luciferase Cell Culture Lysis 5x Reagent per well (Promega). Luciferase activity was quantified using 100 µL of Luciferase Assay Reagent (Promega) as a substrate. Relative luciferase units (RLUs) were measured using a microplate reader (SpectraMax L, Molecular Devices) at 570 nm wave length. The percent neutralization titer for each dilution was calculated as follows: NT = [1−(mean luminescence with immune sera/mean luminescence without immune sera)] × 100. The titers that gave 90% neutralization (NT90) were calculated by determining the linear slope of the graph plotting NT versus serum dilution by using the next higher and lower NT using Office Excel (v2019). In all the experiments, RLU of uninfected cells was measured and was always between 50 and 90.

For the ADE assay, THP1 cells were seeded at a confluency of 2 × 10^6^ cells/mL in a 96-well plate and co-incubated for 48 h with serum samples diluted at 1:5000 or 1:50,000 in the presence of SARS-CoV-2-Spike pseudotyped or VSV-G luciferase lentiviral vector, normalized to 100 ng/mL of p24 antigen. Following incubation, cells were lysed using 100 µL of ONE-Glo Luciferase Assay System per well (Promega). RLUs were measured as above.

### SARS-CoV-2 focus reduction neutralization test

FRNT assay was performed as described recently^55^. Briefly, HeLa-ACE2 cells were seeded in 12 μL of complete DMEM at a density of 2 × 10^3^ cells per well. In a dilution plate, pooled mouse serum was diluted in series with a final volume of 12.5 μL. Then 12.5 μL of SARS-CoV-2 was added to the dilution plate at a concentration of 1.2 × 10^4^ pfu/mL.

After 1 h incubation, the media remaining on the 384-well plate was removed and 25 μL of the virus/serum mixture was added to the 384-well plate. The plate was incubated for 20 h, after which the plate was fixed for 1 h. Each well was then washed three times with 100 μL of 1× PBS, 0.05% Tween; 12.5 μL of human polyclonal sera diluted 1:500 in Perm/Wash buffer (BD Biosciences 554723) were added to each well in the plate and incubated at RT for 2 h. Each well was further washed three times and peroxidase goat anti-human Fab (Jackson Scientific) was diluted 1:200 in Perm/Wash buffer, then added to the plate and incubated at RT for 2 h. The plate was then washed three times and 12.5 μL of Perm/Wash buffer was added to the plate, then incubated at RT for 5 min. The Perm/Wash buffer was removed and TrueBlue peroxidase substrate was immediately added (Sera Care 5510-0030). Sera were tested in triplicate wells. Normal human plasma was used as negative control for serum screening.

### SARS-CoV-2 convalescent plasma samples

UC San Diego Human Research Protections Program (irb.ucsd.edu) approved IBC Protocol 20004 for the use of SARS-CoV-2 convalescent plasma. Anonymized plasma samples of SARS-CoV-2 convalescent individuals (*N* = 19) were obtained from UCSD. The patients gave informed consent. Individuals were confirmed to be infected in the previous 3–10 weeks by PCR and lateral flow assay. All individuals were symptomatic with mild to moderate-severe symptoms. Serum samples (DS-626-G and DS-626-N, Seracare) purchased before SARS-CoV-2 pandemic were used as a negative control. SARS-CoV-2-specific binding antibodies in plasma samples were measured as described above. Cross-adsorbed goat anti-human IgG (H + L) secondary antibody (A18811, Invitrogen) was used at a dilution of 1:3000.

### T-cell analysis

Spleens were harvested and dissociated using a cell mesh following which blood cells were removed using RBC Lysis Buffer (BioLegend). Then 2.5 × 10^6^ splenocytes were stimulated with S or N peptide libraries (GenScript, 15mers with 11aa overlap, 1 µg/mL), 0.1% DMSO, or phorbol myristate acetate (PMA)-ionomycin (BD Biosciences) for 1.5 h at 37 °C. Anti-mouse CD28 and CD49d antibodies (1 µg/mL; BE0328, bioXcell; and 103710, Biolegend, respectively) were added as co-stimulation with the exception of the T-cell analysis of B7 immunized mice. Brefeldin A (3 µg/mL; eBioscience) was added, and the cells were incubated for additional 16 h at 37 °C. Cells were fixed using Cytofix buffer (BD Biosciences) and surface staining was performed using fluorescein isothiocyanate (FITC)-conjugated anti-mouse CD3 (Clone 17A2, 555274, BD), BV650 anti-mouse CD8a (Clone 53-6.7, 563234, BD); PerCP-Cy5.5 anti-mouse CD4 (Clone RM4-5. 550954, BD). Following cell permeabilization using Cytoperm buffer (BD Biosciences), ICS was performed using allophycocyanin (APC)-conjugated anti-mouse IFN-γ (Clone XMG1.2, 554413, BD), phycoerythrin (PE)-conjugated anti-mouse TNF-α (Clone MP6-XT22, 554419, BD), and PE-CF594 anti-mouse IL-2 (BD Biosciences (Clone JES6-5H4, 562483, BD). In experiments testing double recombinants SARS-CoV2 vectors IL-2 antibody was not included and PE-CF594 anti-mouse IL-4 (clone 11B11, 562450, BD) and BV421 rat anti-mouse IL-10 (clone JES5-16E3, 563276, BD) were added. Events were acquired using a BD FACSCelesta flow cytometer (2 × 10^5^ cells/tube) with BD FACSDiva software (v8.0.1.1). Analysis was performed using FlowJo (v10.6.2). Antigen specific T cells were identified by gating on size (FSC vs SSC), doublet negative (FSC-H vs FSC-A), CD3^+^, CD8^+^/CD4^+^. Cytokine positive responses are presented after subtraction of the background response detected in the corresponding unstimulated sample (media added with Brefeldin A 1 h after beginning of mock stimulation) of each individual mouse sample. Polyfunctional T-cells analysis was performed by applying FlowJo Boolean combination gating.

### Cytokine ELISA

Splenocytes (1 × 10^6^) from immunized mice were incubated in v-bottom wells in the presence of 2 µg/mL S or N peptide pools, or without stimulus in a volume of 200 µL; 48 h later, plates were centrifuged at 2000 r.p.m. for 10 min and cell supernatant was collected and stored at −80 °C. Mouse TNF-alpha (MTA00B), Quantikine ELISA kit (R&D systems) was used according to manufacturer’s recommendations.

### IFNγ ELISpot

T-cell detection by IFNγ ELISpot assay was performed according to the manufacturer’s instructions (3321-2A, Mabtech). ELISpot PVDF plates (MSIPS4W10, Millipore) were pre-activated with ethanol and coated with IFNγ-coating antibody. Splenocytes (2 × 10^5^ peptide-stimulated, 2 × 10^4^ PMA/Ionomycin-stimulated) were added to duplicate wells and incubated overnight with 2 µg/mL peptides. Stimuli included S and N peptide libraries; S1 subunit peptide pools covering peptides 1–86 (pool 1S1) and 87–168 (pool 2S1) of the S library; S2 subunit peptide pool that included peptides 169–316 of the S library; and peptide N26 (MKDLSPRWYFYYLGT) of the N peptide library. After 24 h, cells were removed, and IFNγ-detection antibody followed by streptavidin-ALP were added. Spots were developed using BCIP/NBT-plus (3650-10, Mabtech) and analyzed using AID ELISpot reader with AID ELISpot 5.0 iSpot software.

### Statistics

Statistical evaluation was pursued using GraphPad Prism (v8.3.0). For evaluation of differences in sMVA and wtMVA plaque area in BHK-21 and CEF cells and differences in sMVA and wtMVA host cell range, one-way ANOVA followed by Tukey’s and Dunnet’s multiple comparison tests were used, respectively. For sMVA and wtMVA growth kinetic analysis, mixed-effects model with the Geisser-Greenhouse correction followed by Tukey’s multiple comparison test was applied. For ELISAs, one-way ANOVA and Tukey’s multiple comparison tests were used to calculate differences in endpoint titers and group means between groups. For IgG2a/IgG1 ratio and IgG2c/IgG1 ratio analysis, one-way ANOVA with Dunnett’s multiple comparison test was used to compare the ratio measured in each group to a ratio of 1. Pearson correlation analysis was performed to calculate the correlation coefficient *r* and its significance. For T-cell responses analysis, one-way ANOVA followed by Dunnett’s multiple comparison test with a single pooled variance was used to compare the mean of each group. For ELISpot analysis, two-way ANOVA with Dunnett’s multiple comparison test was applied.

### Reporting Summary

Further information on research design is available in the Nature Research Reporting Summary linked to this article.

## Supplementary information

Supplementary Information

Reporting Summary

## Data Availability

The authors declare that all data supporting the findings of the study are available in this article and its Supplementary files, or from the corresponding authors. The sequences of the synthetic constructs that support the findings of this study have been deposited in NCBI with the accession codes MW023923 (sMVA F1), MW023924 (sMVA F2), MW030459 (sMVA F3), MW036243 (sMVA-N/S), and MW030460 (sMVA-S/N). Source data are provided with this paper.

## Source data

Source Data
