# Supplementary material for: Development of a multi-antigenic SARS-CoV-2 vaccine candidate using a synthetic poxvirus platform
Source: Nat Commun. 2020 Nov 30;11:6121. doi: 10.1038/s41467-020-19819-1 (PMC7705736; doi:10.1038/s41467-020-19819-1)
Supplement: Supplementary file 1 — Supplementary Information [file 41467_2020_19819_MOESM1_ESM.pdf]

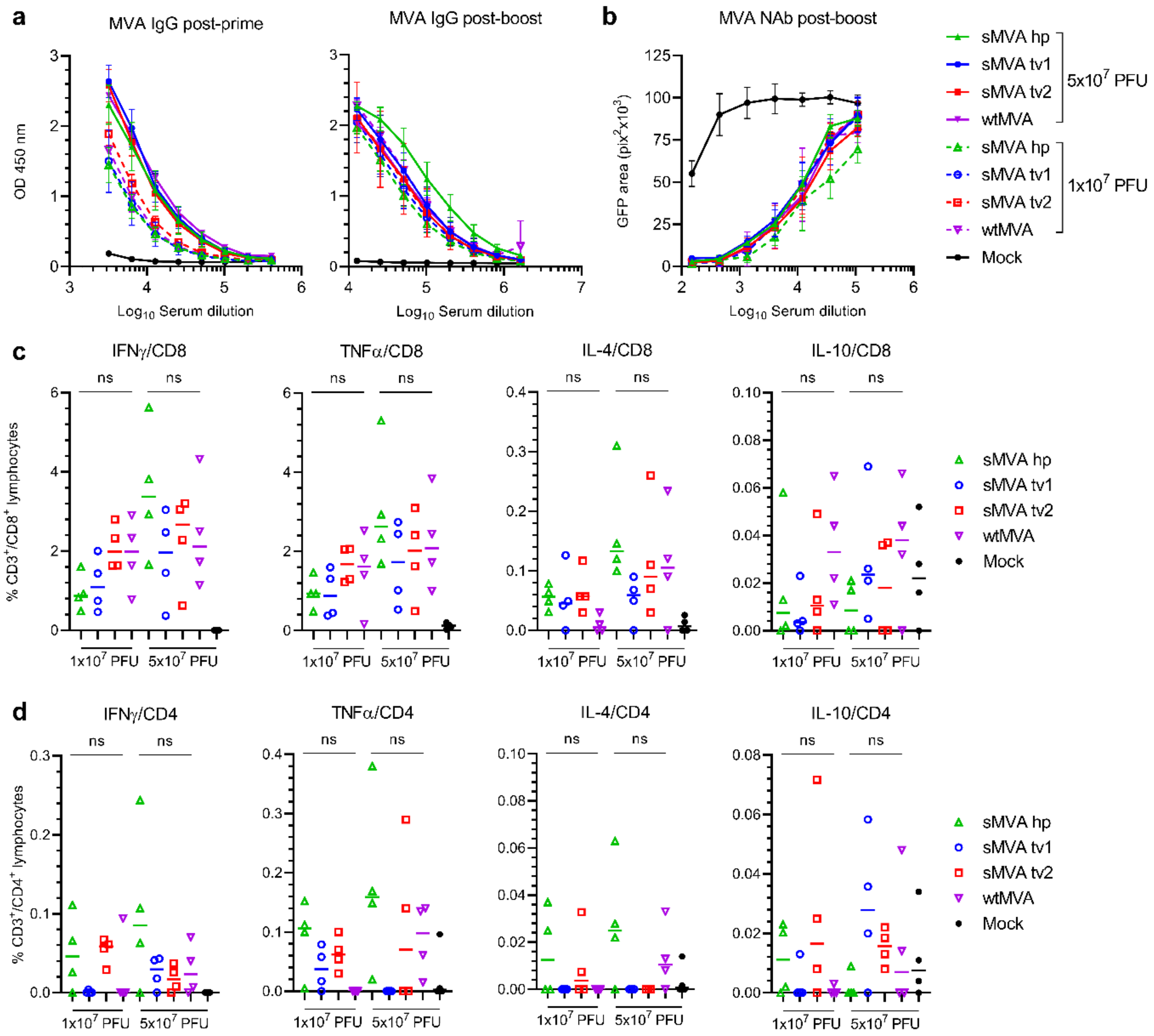

Figure S1. Relative to Figure 3. sMVA immunogenicity in vivo. sMVA derived either with FPV HP1.441 (sMVA hp) or with FPV TROVAC from two independent virus reconstitution (sMVA tv1 and sMVA tv2) was compared by in vivo analysis with wtMVA. C57BL/6 mice (n=4) were immunized twice in a three week interval with low ($1x10^7$ PFU) or high ($5x10^7$ PFU) dose of sMVA or wtMVA. Mock-immunized mice were used as controls **a** Binding antibodies. Shown is the absorbance at 450 nm at different serum dilutions of MVA-specific binding antibodies (IgG titer) measured by ELISA after the first and second immunization in mice receiving sMVA or wtMVA. **b** NAb responses. MVA-specific NAb titers induced by sMVA or wtMVA were measured after the booster immunization against wtMVA expressing a GFP marker. Shown is the measured GFP area of infected cells in square pixels (pix^2x10^3) at different serum dilutions. **c-d** T cell responses. MVA-specific CD8+ (**c**) and CD4+ (**d**) T cells expressing IFNγ, TNFα, IL-4, and IL-10 were measured after two immunizations with sMVA or wtMVA by flow cytometry following ex vivo antigen stimulation using Vaccinia A19L immunodominant peptides. Differences between groups were evaluated using one-way ANOVA with Tukey's multiple comparison test. ns = not significant ($p>0.05$). Data in **a** and **b** are presented as mean values +/- SD. Lines in **c** and **d** represent median values. Source data are provided as a Source Data file.

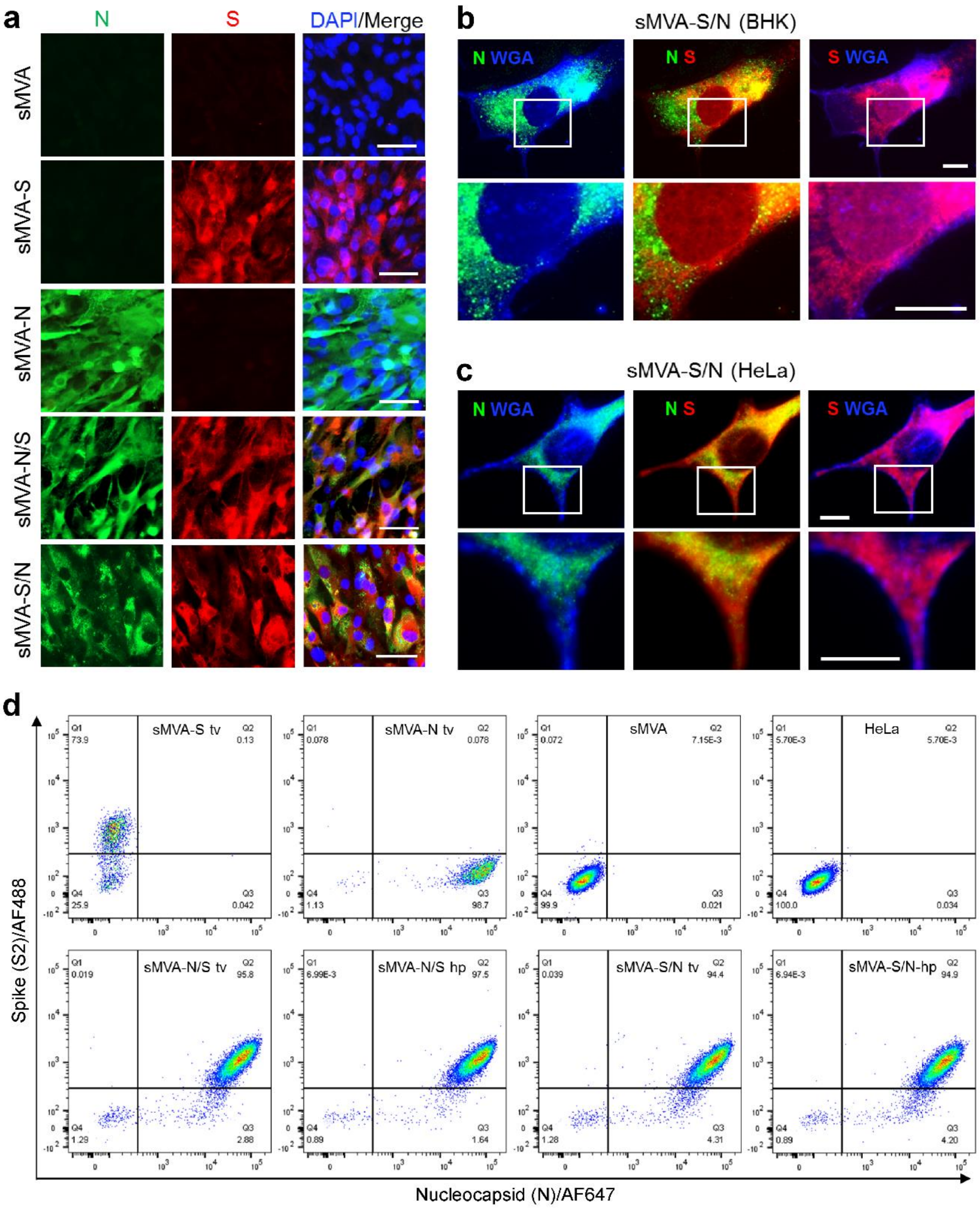

Figure S2. Related to Figure 4. In vitro characterization of sMVA-CoV2 vectors. a-c Immunofluorescence imaging. S and N antigen expression by the single (sMVA-S and sMVA-N) and double (sMVA-S/N and sMVA-N/S) recombinant vaccine sMVA-CoV2 vectors – all derived with FPV TROVAC – was evaluated in BHK (**a** and **c**) or HeLa (**c**) cells by immunofluorescent confocal imaging using N and S-specific antibodies. Fluorescently-conjugated wheat germ agglutinin (WGA) was used in **b** and **c** to stain the cell membrane. Magnified insets are found below images. Scale bars in **a**, 50 μm. Scale bars in **b** and **c**, 10 μm. All images represent two independent experiments with similar results. **d** Flow cytometry dual staining. HeLa cells infected with the single (sMVA-N, sMVA-S) or double (sMVA-N/S, sMVA-S/N) recombinat sMVA-CoV2 vectors derived either with FPV TROVAC (tv) or HP1.441 (hp) were analyszed by intracellular flow cytometry anlayis using dual staining with mouse anti-S2 and rabbit anti-N monoclonal antibodies followed by anti-mouse Alexa Fluor 488 and anti-rabbit Alexa Fluor 647. Percentage of cells dually stained by the S and N-specific antibodies is indicated in the upper-right quadrant (Q2).

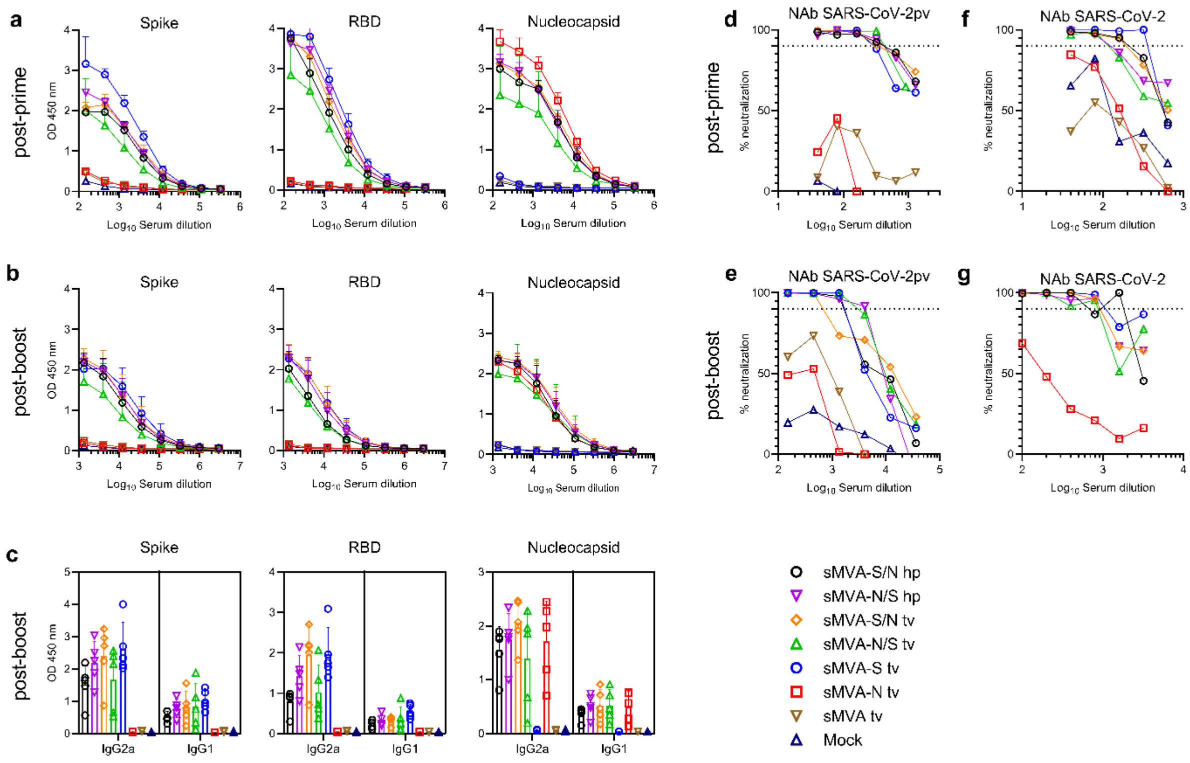

Figure S3. Relative to Figure 5. Humoral immune responses induced by the sMVA-CoV2 vectors. Shown are the antibody measurements in Balb/c mice (n=5) immunized twice in a three week interval with 5x10^{7} PFU of the single or double recombinant sMVA-CoV2 vectors derived with FPV HP1.441 (sMVA-S/N hp and sMVA-N/S hp) or TROVAC (sMVA-S/N tv, sMVA-N/S tv, sMVA-S tv, sMVA-N tv). **a-b** Binding antibodies. Shown are S-, RBD-, and N-specific ELISA measurements at 450 nm using serial dilutions of serum collected two weeks post-prime (**a**) or one-week post-boost (**b**). **c** IgG2a/IgG1 isotype ratio. Binding antibodies of the IgG2a and IgG1 isotypes were measured post-boost in mouse serum using a dilution of 1:10,000. Data in **a**, **b** and **c** is presented as mean values + SD. **d-g** NAb responses. Shown is the percent (%) of SARS-CoV-2pv (**d-e**) and infectious SARS-CoV-2 (**f-g**) neutralization measured in sera pooled from each group of immunized mice. Shown is the average % neutralization in duplicate (**d-e**) or triplicate (**f-g**) infection measured at different serum dilutions. Vaccine groups immunized with sMVA tv and PBS (mock) were not included in the analysis shown in **g** because of failed quality control. Dotted lines mark 90% neutralization that was used to calculate NT90 in Figure 5. Source data are provided as a Source Data file.

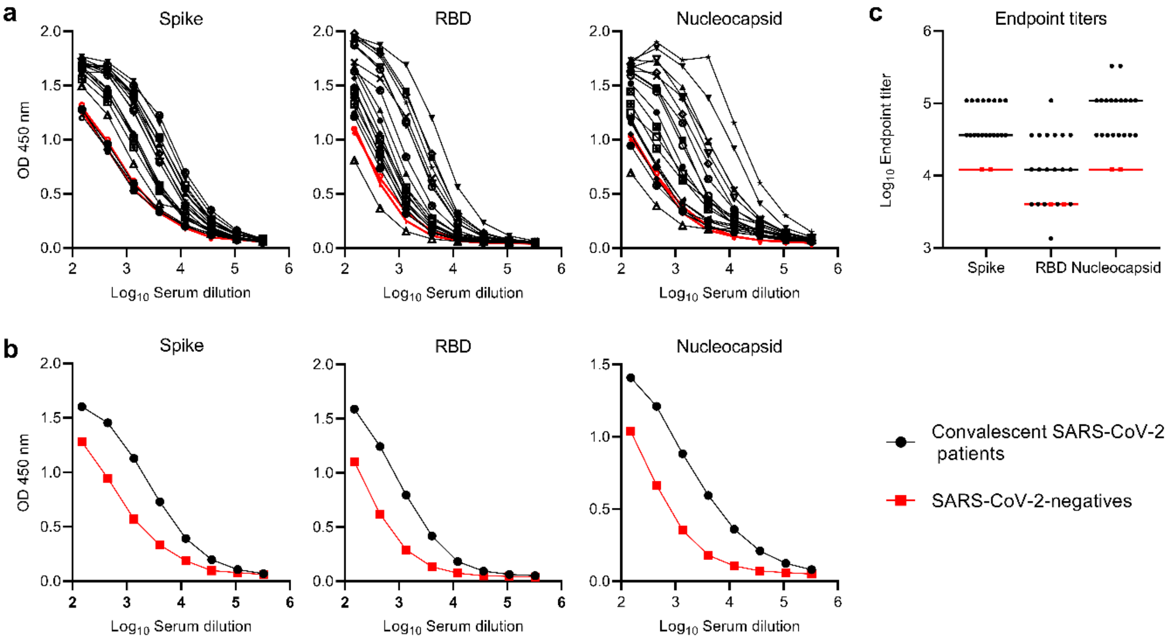

Figure S4. Related to Figure 5. SARS-CoV-2-specific humoral immune responses in convalescent immune sera. S-, RBD, and N-specific binding antibodies were measured via ELISA using serial dilutions of plasma samples from SARS-CoV-2 convalescent individuals. **a** Binding antibody curves from individual samples. **b** SARS-CoV-2 convalescent plasma (n=19) binding curves were grouped and the resulting curve compared to binding measured in samples from SARS-CoV-2 negative individuals (n=2). **c** Endpoint binding antibody titers to S, RBD, and N were calculated in individual plasma samples (n=19 SARS-CoV-2 convalescent individuals, n=2 SARS-CoV-2 seronegative plasma). Lines represent the median endpoint titers. Due to the limited number of SARS-CoV-2-negative samples evaluated, statistical analysis was not performed. Source data are provided as a Source Data file.

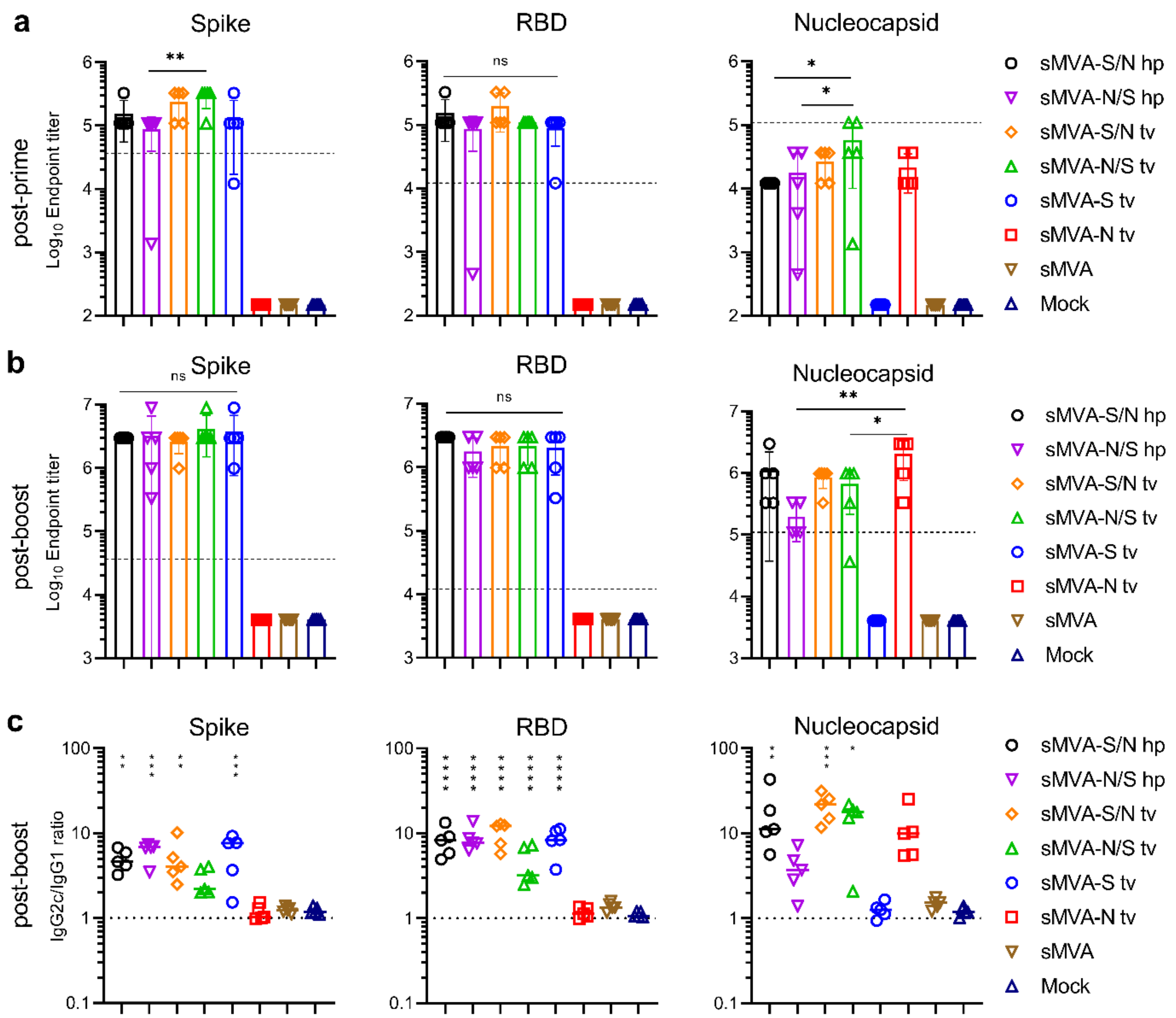

Figure S5. Relative to Figure 5. Humoral immune responses induced by sMVA-CoV2 vectors. C57BL/6 Nramp1 mice (n=5) were immunized twice in a three week interval with $5x10^7$ PFU of the single and double recombinant sMVA-CoV2 vectors derived with FPV HP1.441 (sMVA-S/N hp and sMVA-N/S hp) or TROVAC (sMVA-S/N tv, sMVA-N/S tv, sMVA-S tv, sMVA-N tv) and evaluated for SARS-CoV-2-specific humoral immune responses. **a-b** Binding antibodies. S, RBD, and N-specific binding antibodies induced by the vaccine vectors were evaluated two weeks after the first immunization (**a**) and one week after the second immunization (**b**) by ELISA. Dashed lines in **a** and **b** indicate median binding antibody endpoint titers that were measured in convalescent human sera (Figure S4). Data in **a**, and **b** is presented as mean values +/- SD. One-way ANOVA with Tukey's multiple comparison test was used to compare differences between binding antibody end-point titers in mice immunized with different vaccine vectors. **c** IgG2c/IgG1 isotype ratio. S-, RBD-, and N-specific binding antibodies of the IgG2c and IgG1 isotype were measured after the second immunization using 1:10,000 serum dilution, and absorbance reading was used to calculate IgG2c/IgG1 antibody ratio. One-way ANOVA with Dunnett's multiple comparison test was used to compare each group mean IgG2c/IgG1 ratio to a ratio of 1 (balanced Th1/Th2 response). Lines represent median values. *0.05<p<0.01, **0.01<p<0.001, ***0.001<p<0.0001, ****p<0.0001. ns=not significant. Source data are provided as a Source Data file.

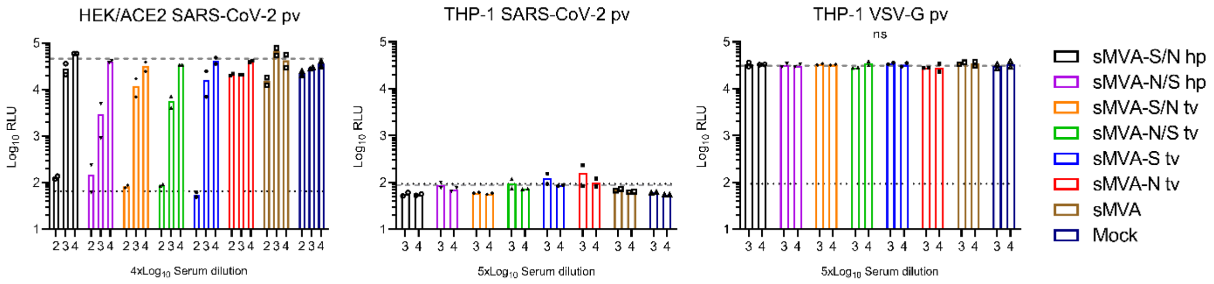

Figure S6. Related to Figure 5. ADE assay. The pooled immune sera of Balb/c mice immunized with $5x10^7$ PFU of the single and double recombinant sMVA-CoV2 vectors derived with FPV HP1.441 (sMVA-S/N hp and sMVA-N/S hp) or TROVAC (sMVA-S/N tv, sMVA-N/S tv, sMVA-S tv, sMVA-N tv) were evaluated for ADE effects. Neutralizing (1:5,000) and non-neutralizing (1:50,000) dilutions (as assayed on stably-transduced HEK293T cells expressing ACE2 (HEK/ACE2)) were evaluated to promote THP-1 monocyte infection by SARS-CoV-2 pseudovirus (pv) expressing luciferase. VSV-G pv was used as infection control. Relative light units (RLU) were measured in duplicates at 48 hours post infection. Dotted lines represent the negative control (average relative light units [RLU] measured in cells in the absence of pv). Dashed lines represent the positive control (average RLU measured in cells in the absence of serum and in the presence of pv). Source data are provided as a Source Data file.

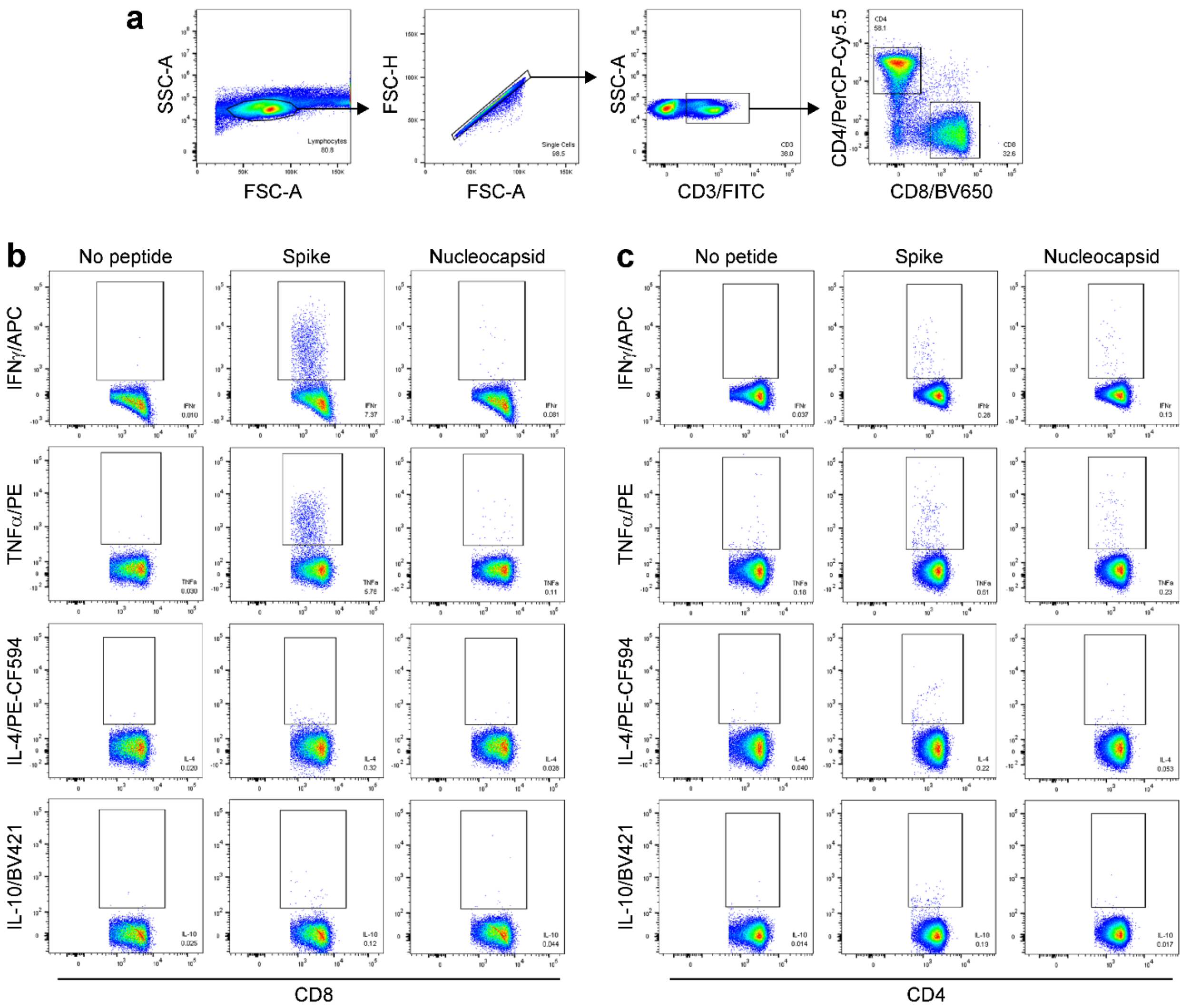

Figure S7. Related to Figure 6. Flow cytometry gating strategy. a Intracellular staining analysis of mouse splenocytes stimulated with S and N peptide libraries was performed using a hierarchical gating strategy that included lymphocytes>singlets>CD3$^+$ T-cells>CD4$^+$ T-cells and CD8$^+$ T-cells>Cytokine positive cells. **b-c** Example of gating on cytokine-positive CD8$^+$ T-cells (**b**) and CD4$^+$ T-cells (**c**). Splenocytes of a mouse immunized with double recombinant vector sMVA-N/S were either left untreated (no peptide) or stimulated 16 hours with S or N peptide pools. Numbers in each dot plot indicate the percentage of cells in gated areas.

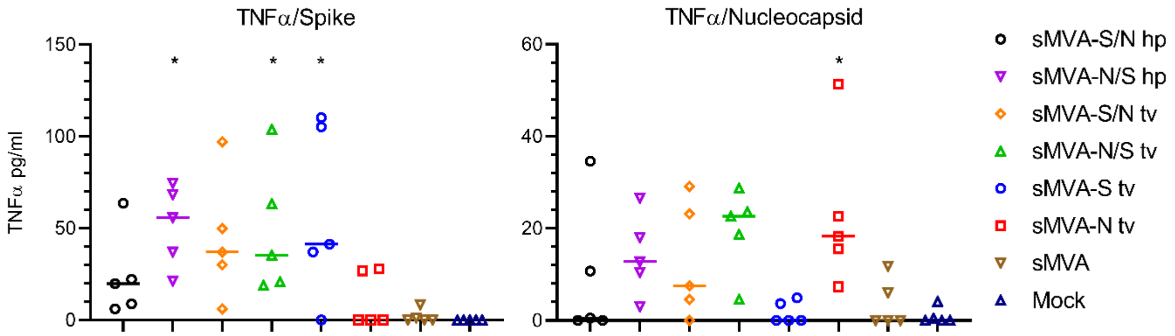

Figure S8. Related to Figure 6. TNFα secretion by T-cells of sMVA-CoV2-immunized mice. Splenoctyes from Balb/c mice (n=5) immunized with $5x10^7$ PFU of the single and double recombinant sMVA-CoV2 vectors derived with FPV HP1.441 (sMVA-S/N hp and sMVA-N/S hp) or TROVAC (sMVA-S/N tv, sMVA-N/S tv, sMVA-S tv, sMVA-N tv) were evaluated for TNFα secretion. Mouse splenocytes were stimulated with S or N peptide libraries and 48 hours later TNFα was measured by ELISA in cell culture supernatants. Amounts of TNFα quantified in unstimulated samples were subtracted from each peptide-stimulated sample. *$0.05<p<0.01$ compared to mock-immunized mice using one-way ANOVA with Dunnett's multiple comparison test. Lines represent median values. Source data are provided as a Source Data file.

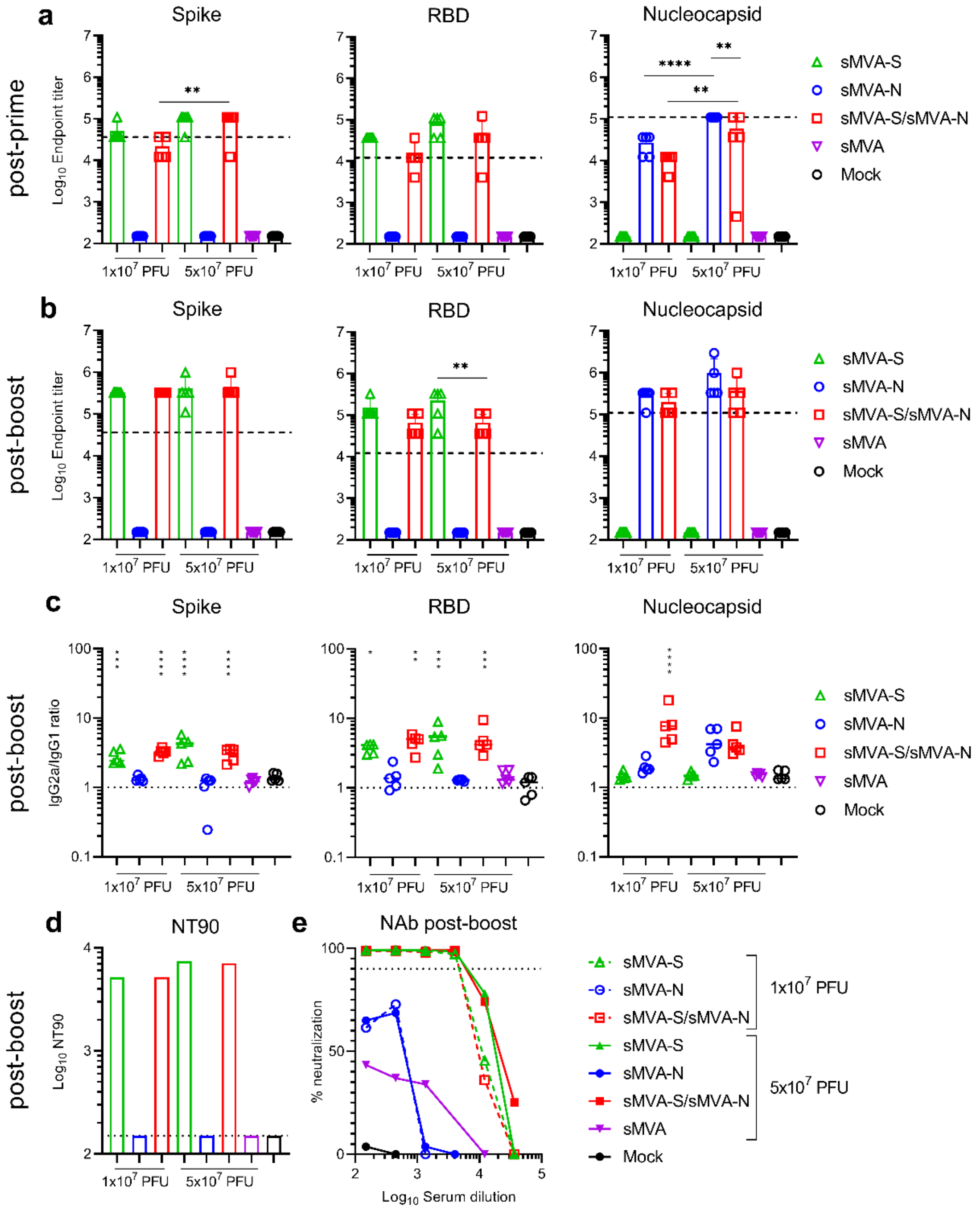

Figure S9. Related to Figure 5. Humoral immune responses induced by sMVA-CoV2 vectors. SARS-CoV-2-specific humoral immune responses were evaluated in mice immunized with the single recombinant vectors sMVA-S and sMVA-N alone or in combination. Balb/c mice (N=5) were immunized twice in three week interval with high ($5x10^7$ PFU) or low ($1x10^7$ PFU) dose of sMVA-S and sMVA-N. Co-immunization via the same immunization schedule with half of the high or low dose of each of the vaccine vectors was evaluated to assess SARS-CoV-2-specific immune stimulation to the S and N antigens by the vectors in combination. Mice immunized with empty sMVA vector or mock-immunized mice were used as controls. **a-b** Binding antibodies. Antigen-specific binding antibodies to S, RBD, and N were determined after the first and second immunization by ELISA. Dashed lines indicate median binding antibody endpoint titers that were measured in convalescent human sera (Figure S4). Data in **a**, and **b** is presented as mean values + SD. One-way ANOVA with Tukey's multiple comparison test was used to compare differences between binding antibody end-point titers in mice immunized with different vaccine doses, and mice immunized with the vaccine vectors alone or combined. **c** IgG2a/IgG1

isotype ratio. Ratio of IgG2a/IgG1 binding antibodies to S, RBD, and N was calculated after performing isotype-specific ELISA for the different antigens using post-boost serum from immunized mice. Lines represent median values. One-way ANOVA with Dunnett's multiple comparison test was used to compare each group mean to a ratio of 1 (balanced Th1/Th2 response). **d-e** NAb titers. SARS-CoV-2-specific NAb responses were measured after the second immunization in pooled sera by neutralization assay using SARS-CoV-2 pseudovirus. Shown in **d** are the neutralizing antibody titers to prevent 90% infection of SARS-CoV-2 pseudovirus (NT90). Dotted baseline represents the minimum dilution included in the analysis. Groups with NT90<baseline are shown at baseline. **e** shows % neutralization measured using serial dilutions of pooled sera. Dotted line in **e** marks 90% neutralization. *0.05<p<0.01, **0.01<p<0.001, ***0.001<p<0.0001, ****p<0.0001. Source data are provided as a Source Data file.

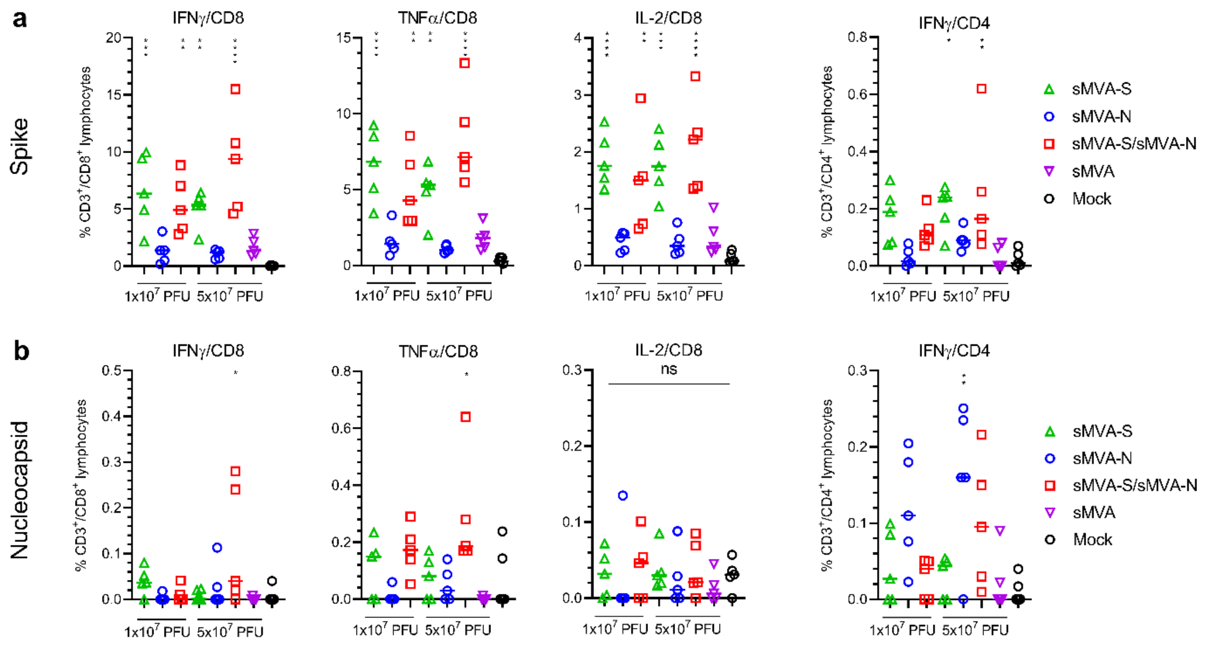

Figure S10. Related to Figure 6. Cellular immune responses *In vivo* immunogenicity of sMVA-CoV2 vectors. a-b SARS-CoV-2-specific cellular immune responses were evaluated in mice immunized with the single recombinant vectors sMVA-S and sMVA-N alone or in combination. Balb/c mice (n=5) were immunized twice in three week interval with high ($5x10^7$ PFU) or low ($1x10^7$ PFU) dose of sMVA-S and sMVA-N. Co-immunization via the same immunization schedule with half of the high or low dose of each of the vaccine vectors was evaluated to assess SARS-CoV-2-specific immune stimulation to the S and N antigens by the vaccine vectors in combination. Mice immunized with empty sMVA vector or mock-immunized mice were used as controls. Antigen-specific CD8+ T cells expressing IFNγ, TNFα, and IL-2 and CD4+ T cell expressing IFNγ were evaluated by flow cytometry staining following *ex vivo* antigen stimulation using SARS-CoV-2-specific S (**a**) and N (**b**) peptide libraries. Lines represent median values. One-way ANOVA followed by Dunnett's multiple comparison test was used to compare each group mean to the mean in mock-immunized mice. *0.05<p<0.01, **0.01<p<0.001, ***0.001<p<0.0001, ****p<0.0001. ns=not significant. Source data are provided as a Source Data file.

Table S1

Construct	Insert (insertion site)	Titer*
sMVA hp	None	$6.8x10^{9}$ PFU/ml
sMVA tv1	None	$4.1x10^{9}$ PFU/ml
sMVA tv2	None	$2.3x10^{9}$ PFU/ml
wtMVA	None	$4.1x10^{9}$ PFU/ml
sMVA-S tv	Spike (Del3)	$4.3x10^{9}$ PFU/ml
sMVA-N tv	Nucleocapsid (Del3)	$1.0x10^{10}$ PFU/ml
sMVA-S/N hp	Spike (G1L), Nucleocapsid (Del3)	$8.8x10^{9}$ PFU/ml
sMVA-N/S hp	Nucleocapsid (Del2), Spike (Del3)	$2.3x10^{9}$ PFU/ml
sMVA-S/N tv	Spike (G1L), Nucleocapsid (Del3)	$8.8x10^{9}$ PFU/ml
sMVA-N/S tv	Nucleocapsid (Del2), Spike (Del3)	$8.4x10^{9}$ PFU/ml

*Stocks were produced on CEF following infection (MOI 0.02) of 30x15cm dishes
