# Supplementary material for: Development of a multi-antigenic SARS-CoV-2 vaccine candidate using a synthetic poxvirus platform
Source: Nat Commun. 2020 Nov 30;11:6121. doi: 10.1038/s41467-020-19819-1 (PMC7705736; doi:10.1038/s41467-020-19819-1)
Supplement: Supplementary file 2 — Reporting Summary [file 41467_2020_19819_MOESM2_ESM.pdf]

nature research

Corresponding author(s): Felix Wussow
Don J. Diamond

Last updated by author(s): Oct 27, 2020

Reporting Summary

Nature Research wishes to improve the reproducibility of the work that we publish. This form provides structure for consistency and transparency in reporting. For further information on Nature Research policies, see our Editorial Policies and the Editorial Policy Checklist.

Statistics

For all statistical analyses, confirm that the following items are present in the figure legend, table legend, main text, or Methods section.

n/a	Confirmed	
☐	☒	The exact sample size (n) for each experimental group/condition, given as a discrete number and unit of measurement
☐	☒	A statement on whether measurements were taken from distinct samples or whether the same sample was measured repeatedly
☐	☒	The statistical test(s) used AND whether they are one- or two-sided<br>*Only common tests should be described solely by name; describe more complex techniques in the Methods section.*
☒	☐	A description of all covariates tested
☐	☒	A description of any assumptions or corrections, such as tests of normality and adjustment for multiple comparisons
☐	☒	A full description of the statistical parameters including central tendency (e.g. means) or other basic estimates (e.g. regression coefficient) AND variation (e.g. standard deviation) or associated estimates of uncertainty (e.g. confidence intervals)
☐	☒	For null hypothesis testing, the test statistic (e.g. F, t, r) with confidence intervals, effect sizes, degrees of freedom and P value noted<br>*Give P values as exact values whenever suitable.*
☒	☐	For Bayesian analysis, information on the choice of priors and Markov chain Monte Carlo settings
☒	☐	For hierarchical and complex designs, identification of the appropriate level for tests and full reporting of outcomes
☐	☒	Estimates of effect sizes (e.g. Cohen's d, Pearson's r), indicating how they were calculated

Our web collection on statistics for biologists contains articles on many of the points above.

Software and code

Policy information about availability of computer code

Data collection	BD FACSDiva (v8.0.1.1); Zen software (Zeiss, Black Edition Version 8.1); LAS X (v2.0.0.14332); SoftMax Pro (v7); Syngene Pxi6; AID ELISpot 5.0 iSpot
Data analysis	GraphPad Prism 8 (v8.3.0); Office Excel (v2019); FlowJo (v10.6.2); SMRT Portal (v. 2.3.0); SMRT Link (v6.0.0.47841); SMRT Link (v8.0.0.80529); VarScan (v2.3.9); pbmm2v 1.0.0; canu (v1.7.1); LAS X s(v2.0.0.14332); Image-Pro Premier (v9.2); GeneSys (v1.5.4.0); Vector NTI (v11.5)

For manuscripts utilizing custom algorithms or software that are central to the research but not yet described in published literature, software must be made available to editors and reviewers. We strongly encourage code deposition in a community repository (e.g. GitHub). See the Nature Research guidelines for submitting code & software for further information.

Data

Policy information about availability of data

All manuscripts must include a data availability statement. This statement should provide the following information, where applicable:

- Accession codes, unique identifiers, or web links for publicly available datasets
- A list of figures that have associated raw data
- A description of any restrictions on data availability

The authors declare that all data supporting the findings of the study are available in this article and its Supplementary files, or from the corresponding authors. The source data are provided with this paper. The sequences of the synthetic constructs that support the findings of this study have been deposited in NCBI with the accession codes MW023923 (sMVA F1), MW023924 (sMVA F2), MW030459 (sMVA F3), MW036243 (sMVA-N/S) and MW030460 (sMVA-S/N).

Field-specific reporting

Please select the one below that is the best fit for your research. If you are not sure, read the appropriate sections before making your selection.

☒ Life sciences ☐ Behavioural & social sciences ☐ Ecological, evolutionary & environmental sciences

For a reference copy of the document with all sections, see nature.com/documents/nr-reporting-summary-flat.pdf

Life sciences study design

All studies must disclose on these points even when the disclosure is negative.

Sample size	Sample size was selected based upon mean and standard deviations observed in our previous vaccine studies in these animal models, and ability to detect statistical differences between experimental groups of this size (https://doi.org/10.1128/JVI .01012-18). Additionally, previous reports relevant to our study have used similar group sizes with comparable significance of the results (https://doi.org/10.1038/s41467-020-16505-0; https://doi.org/10.1101/2020.07.29.227595).
Data exclusions	All data were included in the analysis with the exception of:<br>Figure 5G - SARS-CoV-2 FRNT assay. Control samples sMVA and Mock failed quality control.<br>Figure 6C-G- CD4/TNFa ICS- 1-3 animals/group were excluded from the analysis due to a technical issue.<br>Data exclusion for the above is indicated in the figure legend.
Replication	sMVA replication kinetics were performed using triplicate infections for each time point. Input virus titer was calculated from 2 infections. CEF, BHK and different human cell lines for the host range analysis were infected in duplicates and the virus titers for each duplicate infection was titrated in duplicates (total n=4). sMVA replication kinetics and host range analysis were performed once.<br>PCR analysis of sMVA vectors and sMVA-CoV2 vectors using infected CEF was performed twice with similar results.<br>Western Blot analysis of the the sMVA-CoV2 antigen expression was performed twice using BHK cells and repeated on CEF with similar results.<br>Cytofluorimetric analysis of cells infected with sMVA recombinants was repeated twice using both HeLa and HEK293 cells with similar results.<br>Immunofluorescence staining of SARS-CoV-2 antigen expression by the vaccine vectors was repeated twice with similar results.<br>Assays involving fresh mouse splenocytes (ICS, ELISPOT) were not repeated due to the impossibility of cryopreserving mouse splenocytes and their limited amount.<br>Antibody analyses were performed once given the limited amount of serum allowed to be withdrawn from each animal and required across multiple assays (ELISAs for S, RBD, N total IgG, IgG1 and IgG2a/c; neutralization assays with live virus and pseudovirus, ADE assay).<br>Neutralization assays and ELISPOT were performed in duplicate/triplicate wells and results averaged.
Randomization	For experiments involving mice, animals of similar age were randomly assigned to each group. When both male and female animals were used, numbers of animals of each sex were balanced amongst groups.
Blinding	Operators performing FRNT assay, SARS-CoV-2 pseudovirus assay, and ADE assay were blinded to the samples identity.<br>Operators performing remaining assays were not blinded to the sample's identity given their involvement in both sample preparation and analysis.

Reporting for specific materials, systems and methods

We require information from authors about some types of materials, experimental systems and methods used in many studies. Here, indicate whether each material, system or method listed is relevant to your study. If you are not sure if a list item applies to your research, read the appropriate section before selecting a response.

Materials & experimental systems

n/a	Involved in the study	
☐	☒	Antibodies
☐	☒	Eukaryotic cell lines
☒	☐	Palaeontology and archaeology
☐	☒	Animals and other organisms
☐	☒	Human research participants
☒	☐	Clinical data
☒	☐	Dual use research of concern

Methods

n/a	Involved in the study	
☒	☐	ChIP-seq
☐	☒	Flow cytometry
☒	☐	MRI-based neuroimaging

Antibodies

Antibodies used	anti-SARS-CoV-1 S1 subunit rabbit polyclonal antibody (40150-T62-COV2, Sino Biological); anti-SARS-CoV1 NP rabbit polyclonal antibody (40413-T62, Sino Biological); anti vaccinia BR5 rat monoclonal antibody 19c2 (Schmelz et al.), Anti-rabbit IgG polyclonal antibody (A6154, Sigma-Aldrich); anti-rat IgG polyclonal antibody (A5795, Sigma-Aldrich); Anti-SARS-CoV-1 S1 rabbit (40150-R007, Sino Biological); anti-SARS-CoV-1/CoV-2 S2 mouse (clone 1A9; GTX632604, GeneTex); anti-SARS-CoV-1 N rabbit monoclonal antibody

(40143-R001, Sino Biological); anti-vaccinia rabbit polyclonal antibody (9503-2057, Bio Rad); anti-mouse Alexa Fluor 488 (A11001; Invitrogen); anti-rabbit Alexa Fluor 488 (A21206; Invitrogen); anti-rabbit Alexa Fluor 647 (A21244; Invitrogen); Alexa Fluor 350 Conjugated Wheat Germ Agglutinin (W11263, Invitrogen); HRP-conjugated anti-mouse IgG secondary antibody (W402B, Promega); goat Anti-Mouse IgG2a cross absorbed HRP antibody, Southern biotech, 1083-05; Goat anti-Mouse IgG1 cross absorbed HRP antibody (Thermo Fisher, A10551) ; Goat anti-mouse IgG2c HRP antibody, Thermo Fisher, PA1-29288); Cross-adsorbed goat anti-human IgG (H+L) secondary antibody (A18811, Invitrogen); LEAF Purified anti-mouse CD49d Antibody (103710 , Biolegend); InVivoMAb anti-mouse CD28 (BE0328, bioxcell); fluorescein isothiocyanate (FITC)-conjugated anti-mouse CD3 (Clone 17A2, 555274, BD), PerCP-Cy5.5 anti-mouse CD4 (Clone RM4-5. 550954, BD), BV650 anti-mouse CD8a (Clone 53-6.7, 563234, BD). Following cell permeabilization using Cytoperm buffer (BD Biosciences), ICS was performed using allophycocyanin (APC)-conjugated anti-mouse IFN-γ (Clone XMG1.2, 554413, BD), phycoerythrin (PE)-conjugated anti-mouse TNF-α (Clone MP6-XT22, 554419, BD), and PE-CF594 anti-mouse IL-2 (BD Biosciences (Clone JES6-5H4, 562483, BD); PE-CF594 anti-mouse IL-4 (clone 11B11, 562450, BD) and BV421 rat anti mouse IL-10 (clone JES5-16E3, 563276, BD); Serum samples (DS-626-G and DS-626-N, Seracare).

Validation

Anti-SARS-CoV-1 S1 subunit rabbit polyclonal antibody (40150-T62-COV2, Sino Biological). Validated by manufacturer using SARS-CoV-2 (2019-nCoV) Spike S1 (Cat# 40591-V08H). Validated in house using S1+S2, 40589-V08B1, Sino Biological.
Anti-SARS-CoV1 NP rabbit polyclonal antibody (40413-T62, Sino Biological); Validated by manufacturer using SARS-CoV-2 (2019-nCoV) NP protein (Cat# 40588-V08B). Validated in house using the same protein (40588-V08B).
Anti-vaccinia BR5 rat monoclonal antibody (Schmelz et al.). Used in Chiuppesi, F. et al. (2018), Multiantigenic Modified Vaccinia Virus Ankara Vaccine Vectors To Elicit Potent Humoral and Cellular Immune Reponses against Human Cytomegalovirus in Mice. Journal of Virology. https://doi.org/10.1128/JVI .01012-18.
Anti-SARS-CoV-1 S1 rabbit (40150-R007, Sino Biological). Validated by manufacturer using HEK293T/Ace2 cells infected with SARS-CoV-2 spike pseudovirus.
Anti-SARS-CoV-1/CoV-2 S2 rabbit (clone 1A9; GTX632604, GeneTex). Validated by vendor using Caco-2 cells infected with SARS-CoV-2.
Anti-SARS-CoV-1 N rabbit monoclonal antibody (40143-R001, Sino Biological). Validated by manufacturer using SARS-CoV-2 (2019-nCoV) NP protein (Cat# 40588-V08B).
Anti-vaccinia rabbit polyclonal antibody (9503-2057, Bio Rad). Validated using vaccinia purified virions. Used in Manuel, E.R. et al. (2010) Intergenic region 3 of modified vaccinia ankara is a functional site for insert gene expression and allows for potent antigen-specific immune responses. Virology. 2010 403: 155-62.
Fluorescein isothiocyanate (FITC)-conjugated anti-mouse CD3 (Clone 17A2, 555274, BD). Validated by vendor using mouse thymocytes and splenocytes.
PerCP-Cy5.5 anti-mouse CD4 (Clone RM4-5. 550954, BD). Validated by vendor using mouse splenocytes.
BV650 anti-mouse CD8a (Clone 53-6.7, 563234, BD). Validated by vendor using mouse splenocytes.
allophycocyanin (APC)-conjugated anti-mouse IFN-γ (Clone XMG1.2, 554413, BD). Validated by vendor using mouse splenocytes.
phycoerythrin (PE)-conjugated anti-mouse TNF-α (Clone MP6-XT22, 554419, BD). Validated by vendor using mouse splenocytes.
PE-CF594 anti-mouse IL-2 (BD Biosciences (Clone JES6-5H4, 562483, BD. Validated by vendor using mouse splenocytes.
anti-mouse CD3, anti-mouse CD4, anti-mouse CD8a, anti-mouse IFN-γ, anti-mouse TNF-α, and anti-mouse IL-2 were used in Chiuppesi, F. et al. (2018); Multiantigenic Modified Vaccinia Virus Ankara Vaccine Vectors To Elicit Potent Humoral and Cellular Immune Reponses against Human Cytomegalovirus in Mice. Journal of Virology. https://doi.org/10.1128/JVI .01012-18.
PE-CF594 anti-mouse IL-4 (clone 11B11, 562450, BD). Validated by vendor using mouse splenocytes.
BV421 rat anti mouse IL-10 (clone JES5-16E3, 563276, BD); Validated by vendor using mouse splenocytes.

Eukaryotic cell lines

Policy information about cell lines

Cell line source(s)	BHK-21 (CCL-10), A549 (CCL-185), HeLa (CCL-2), 293T (CRL-1573), 143B (CRL-8303), HEK293/17 (CRL11268), THP-1 (TIB-202), ARPE-19 (CRL-2302) all from ATCC. CEF were purchased from Charles River (10100795). HEK293T/ACE2 were a gift from Pamela J. Bjorkman and were developed by Jesse D. Bloom (https://doi.org/10.3390/v12050513).
Authentication	All cell lines were purchased (except for HEK293T/ACE2). None of the cell lines were authenticated in house.
Mycoplasma contamination	Cell lines were not tested for mycoplasma contamination
Commonly misidentified lines (See ICLAC register)	No commonly misidentified lines were used

Animals and other organisms

Policy information about studies involving animals; ARRIVE guidelines recommended for reporting animal research

Laboratory animals	6 weeks old female C57BL/6 (C57BL/6J, 000664) or female Balb/c (BALB/cJ, 000651) were purchased from the Jackson laboratories. 6-9 weeks old female and male C57BL/6 Nramp were bred at the City of Hope animal facility. 7 weeks old female and male HLA-B*0702 H-2KbDb double-knockout (B7) transgenic mice on a C57BL/6 background were obtained from F. Lemonnier (Institut Pasteur, France) and bred at the City of Hope Animal Research Center.
Wild animals	No wild animals were used in the study
Field-collected samples	No field collected samples were used in the study
Ethics oversight	The Institutional Animal Care and Use Committee (IACUC) of the Beckman Research Institute of City of Hope (COH) approved protocol 20013 assigned for this study. All study procedures were carried out in strict accordance with the recommendations in the

Guide for the Care and Use of Laboratory Animals and the Public Health Service Policy on the Humane Care and Use of Laboratory Animals

Note that full information on the approval of the study protocol must also be provided in the manuscript.

Human research participants

Policy information about studies involving human research participants

Population characteristics	Female and male individuals aged 20-72. SARS-CoV-2 convalescent patients with mild to moderate-severe symptoms. In most cases diagnosis was confirmed by PCR and/or lateral flow assay. Plasma was collected at 21-67 days post symptoms onset.
Recruitment	Anonymized plasma samples of SARS-CoV-2 convalescent individuals (N=19) were obtained from UCSD. Self-selection bias may be a factor given that very sick people might have been be less likely to participate. Selection bias may have been a factor since volunteers were recruited through personal referrals and by advertising the study on a local newspaper so not thorough targeting of low socio-economic status people or various literacies.
Ethics oversight	UC San Diego Human Research Protections Program (irb.ucsd.edu) approved IBC Protocol 20004 for the use of SARS-CoV-2 convalescent plasma.

Note that full information on the approval of the study protocol must also be provided in the manuscript.

Flow Cytometry

Plots

Confirm that:

- [x] The axis labels state the marker and fluorochrome used (e.g. CD4-FITC).
- [x] The axis scales are clearly visible. Include numbers along axes only for bottom left plot of group (a 'group' is an analysis of identical markers).
- [x] All plots are contour plots with outliers or pseudocolor plots.
- [x] A numerical value for number of cells or percentage (with statistics) is provided.

Methodology

Sample preparation	HeLa cells were seeded in a 6-well plate (5x10^5/well) and infected the following day with sMVA vaccine candidates at an MOI of 5. Following an incubation of 6 hours, cells were detached with non-enzymatic cell dissociation buffer (13151014, GIBCO). Cells were either incubated directly with primary antibody or fixed and permeabilized prior to antibody addition. Primary antibodies were used in dilution 1:2,000. One hour later, secondary antibodies were added at 1:4,000 dilution. Live cells were ultimately fixed with 1% paraformaldehyde (PFA).<br>Spleens were harvested and dissociated using a cell mesh following which blood cells were removed using RBC Lysis Buffer (BioLegend). 2.5x106 splenocytes were stimulated with S or N peptide libraries (GenScript, 15mers with 11aa overlap, 1µg/ml), 0.1% DMSO, or phorbol myristate acetate (PMA)-ionomycin (BD Biosciences) for 1.5 h at 37°C. Anti-mouse CD28 and CD49d antibodies (1µg/ml; BioLegend) were added as co-stimulation with the exception of the T cell analysis of B7 immunized mice. Brefeldin A (3µg/ml; eBioscience) was added, and the cells were incubated for additional 16 h at 37°C. Cells were fixed using Cytofix buffer (BD Biosciences) and surface staining was performed. Cells were washed and intracellular cytokine staining performed. 2x10^5 cells/tube were acquired.
Instrument	BD FACSCelesta flow cytometer
Software	BD FACSDiva software to acquire data. FlowJo v10.6.2 to analyze data.
Cell population abundance	No sorting performed
Gating strategy	Antigen specific T cells were identified by gating on size (FSC vs SSC), doublet negative (FSC-H vs FSC-A), CD3+, CD8+/CD4+.

- [x] Tick this box to confirm that a figure exemplifying the gating strategy is provided in the Supplementary Information.
